# Microwave-Driven Cytocompatible Mn-Doped TiO_2_–Fe_3_O_4_ Ordered Heterostructures for Microplastic and Antibiotic Degradation

**DOI:** 10.1021/acsami.5c20671

**Published:** 2026-02-04

**Authors:** Anjali Valadi Palliyalil, Aiswarya Vijayakumar Thelappurath, Daniel Wojtas, Naděžda Pizúrová, Monika Pávková Goldbergová, Sanjay Gopal Ullattil

**Affiliations:** † Institute of Physics of Materials, Czech Academy of Sciences, Žižkova 513/22, 616 00 Brno, Czech Republic; ‡ Central European Institute of Technology—Brno University of Technology, Purkyňova 656/123, 612 00 Brno, Czech Republic; § Department of Pathophysiology, Faculty of Medicine, 37748 Masaryk University, Kamenice 753/5, 625 00 Brno, Czech Republic

**Keywords:** ordered heterostructure, osteoblasts, cytotoxicity, microplastic degradation, antibiotic degradation

## Abstract

Precisely assembling diverse nanocrystals is crucial for fabricating advanced heterostructures with complex functionalities, collective properties, and enhanced stability, which are essential for addressing widespread pollutants, such as omnipresent microplastics and antibiotics. Achieving photoactive designs for these materials with low cytotoxicity using rapid one-pot methods remains a significant challenge. Herein, we developed a microwave synthesis strategy for Mn-doped TiO_2_–Fe_3_O_4_ ordered heterostructures (TFM) achieved through polyvinylpyrrolidone (PVP) templating. The rapid microwave heating facilitates both swift nucleation and growth, while PVP’s varied affinities for Ti^4+^, Fe^3+^, and Mn^2+^ ions promote oriented attachment and the formation of ordered heterostructures. The resultant heterostructures, characterized by coherently aligned nanocrystals, exhibit significantly enhanced photocatalytic activity, as evidenced by their effective photofragmentation of polyethylene glycol microplastic and tetracycline antibiotic. Prior to photocatalysis, cytotoxicity assessments conducted with osteoblast cells confirm the biocompatibility of these materials, suggesting preliminary potential for environmentally relevant applications, provided short-term TFM exposure is considered. This work, therefore, introduces a versatile and rapid fabrication approach for mesocrystal-inspired heterostructures, underscoring their dual role in pollutant remediation and the development of biocompatible materials, thereby bridging the gap between sustainable synthesis and functional application within the field.

## Graphical abstract

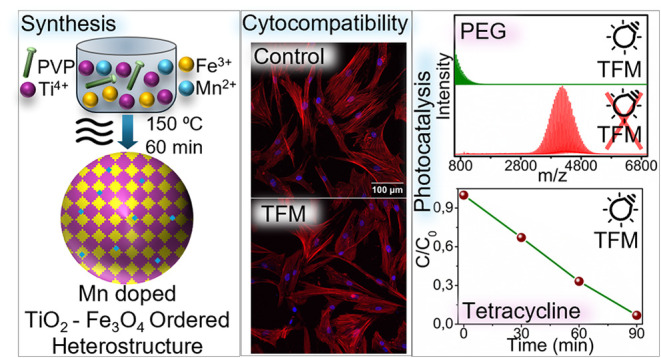

## 1 Introduction

Heterostructures are well-defined semiconductor structures formed by combining different materials, and the resultant structure possesses multifunctional physicochemical properties inherited from the constituent nanomaterials.^1^ The characteristics of the ordered heterostructure will be closely related to the crystal phase, shape, and crystallographic orientation of the subunits of the different nanomaterials constituting the heterostructure. The inherent subunits undergo self- and/or directed assembly to form an ordered heterostructure with a better electronic structure and optimized interfaces.^2^ Unlike randomly aggregated heterostructures, ordered heterostructures have better interfaces with dynamic synergistic effects resulting from the lattice orientation of the nanosubstituents, promoting enhanced mass diffusion and charge transfer, eventually resulting in faster reaction kinetics. Such improved functionalities highlight their application potential in photocatalysis, a viable strategy for environmental remediation.^2^ There are reported studies that reveal the potential of ordered heterostructures as photocatalysts with strong absorption capability and enhanced charge transfer and separation of photogenerated charge carriers.^3,4^ Despite these fascinating features, synthesis of ordered heterostructures remains challenging, since the spatial orientation and controlled alignment of the inherent subunits are often challenging. Moreover, precise control over the crystallization process during the heterostructure formation is necessary since the different nanomaterials have differing formation mechanisms and kinetics.

TiO_2_ is one of the unequivocally accepted parent materials in the heterostructures owing to its strong oxidation power, robustness, and chemical inertness.^5^ As a wide-gap semiconductor with a band gap energy of 3.2 eV, TiO_2_ has a major drawback that limits its photocatalytic activity, due to the faster recombination of charge carriers and low quantum efficiency.^5^ Construction of heterostructures is a better approach to resolving this issue. Incorporation of magnetic nanomaterials such as magnetite comes in handy in this case since these metal oxides have better chemical, optical, and thermal properties with a narrow band gap (1.98 eV).^6^ Thus, incorporation of magnetite as a secondary component into the TiO_2_ matrix helps in tuning the band gap of TiO_2_ without compromising the photocatalytic performance.

Earlier reports suggest that Fe_3_O_4_/TiO_2_ heterostructures effectively promote the separation of photocatalytically generated charge carriers and improve the interfacial electron transfer through the redox cycling of Fe^3+^/Fe^2+^ in Fe_3_O_4_, promoting increased reactive oxygen species (ROS) production and leading to improved photocatalytic activity compared to bare TiO_2_ nano/microarchitectures.^7,8^ The recent literature suggests that the involvement of Mn ions in tuning the band structure is feasible for photocatalysis.^9,10^ Integration of manganese ions into the photocatalytic systems becomes advantageous since they have better redox properties and flexibility in switching the valence states. Thus, they could actively participate in generating reactive species by undergoing multivalent redox transitions.^10^ Such binary and ternary heterostructures have been synthesized using various methods, such as hydrothermal,^8^ sol–gel,^11^ and microwave methods.^12^ However, the microwave method offers rapid and homogeneous heating and provides significant advantages in terms of energy, time, and structural control and thereby depicts a more efficient, faster method in contrast to the conventional methods.^13^

Beyond photocatalytic performance, the investigation of cytocompatibility of heterostructures is necessary for assessing the potential impact of these synthesized materials on biological systems. The evaluation of biocompatibility of the ordered heterostructures is a crucial step to study how the system affects the cell growth, metabolism, and immune system in the organism, thereby ensuring their secure and practical applications in biomedical and clinical trials. TiO_2_ in its pristine and modified forms are widely used in biomedical research due to its biocompatibility, high chemical stability, and low toxicity.^14^ Therefore, it has been widely used in bone tissue engineering^15^ and is a favored material for dental^16^ and orthopedic implants.^17^ TiO_2_ nanoparticles are relevant in bone cell research as their cytotoxic evaluation is conducted using osteoblasts, the cells which are responsible for the formation and mineralization of bone tissue.^18^ Osteoblasts are cuboidal cells with a spherical nucleus that play the key role in synthesizing and maintaining the skeletal framework. TiO_2_ nanoparticles, due to their excellent cytocompatibility, tend to promote osteoblast cell adhesion and proliferation and encourage the maturation and development of bone-forming cells.^19^ Incorporation of iron species into the TiO_2_ lattice can enhance the proliferation and cell differentiation and adhesion of osteoblasts and bone marrow stem cells,^20^ even though iron species alone exhibit cytotoxic effects and often disrupt cellular functions. Upon incorporation along with TiO_2_, its cytotoxicity was reduced markedly and showed good overall biocompatibility, thus enabling the material to be employed in biomedical applications. Manganese has shown positive effects on material biocompatibility, acting not only as a noncytotoxic dopant but also as a bioactive stimulant that enhances osteoblast viability and differentiation. When incorporated into the hydroxyapatite matrix, it supported osteoblast adhesion, migration, and proliferation.^21,22^ More recently, rhombic-shaped Mn-doped TiO_2_ was showcased as a potential magnetic resonance imaging (MRI) agent and a therapeutic agent.^23^ The presence of manganese ions in trace amounts also enhances the functionality of TiO_2_ with an increased osteoblastic cell response due to their cell adhesion-promoting effect and enhances the viability and proliferation of bone cells.^24^ Such materials with known cytotoxicity results can be safely implemented in environmental remediation given their low cytotoxicity.

The ubiquitous and substantial presence of microplastics (<5 mm in size) and antibiotic residues in the aquatic environment poses a serious threat to the environment and human health.^25^ White pollution, a term recently coined to describe the marine environment contaminated with plastics, has emerged as a significant concern in contemporary discussions.^26^ Microplastics in the human body pose serious health risks, which include immune modulation, cardiovascular events, and even reproductive health impairment.^27^ In this context, polyethylene glycol (PEG) is considered a skeptical microplastic widely used in cosmetics, pharmaceuticals, and industries.^28^ In addition to the increased usage of cosmetics containing PEG in daily life, antibiotic usage is also a prime concern, notably one of the widely used antibiotics, tetracycline (TC), in pharmaceuticals, agriculture, and personal care products, positioning it as an emerging organic pollutant. This broad-spectrum antibiotic, being highly soluble, accumulates in sources and causes adverse health effects in humans. Moreover, persistence of these antibiotic residues leads to the development of antibiotic-resistant genes and antibiotic-resistant bacteria.^29^ Hence, a viable option, such as photocatalysis, can address the growing concern over omnipresent microplastics and antibiotics.

Herein, we have developed a one-pot microwave synthesis method for Mn-doped TiO_2_–Fe_3_O_4_ ordered heterostructures (TFM) with surface defects. Their cytotoxicity has been assessed using osteoblast bone cells, and due to their known lower cytotoxicity, TFM has been applied in photocatalysis for the photofragmentation of a microplastic model system, PEG, and an antibiotic model system, tetracycline. A schematic illustration outlining the current study is shown in Scheme 1.

**Scheme 1:**
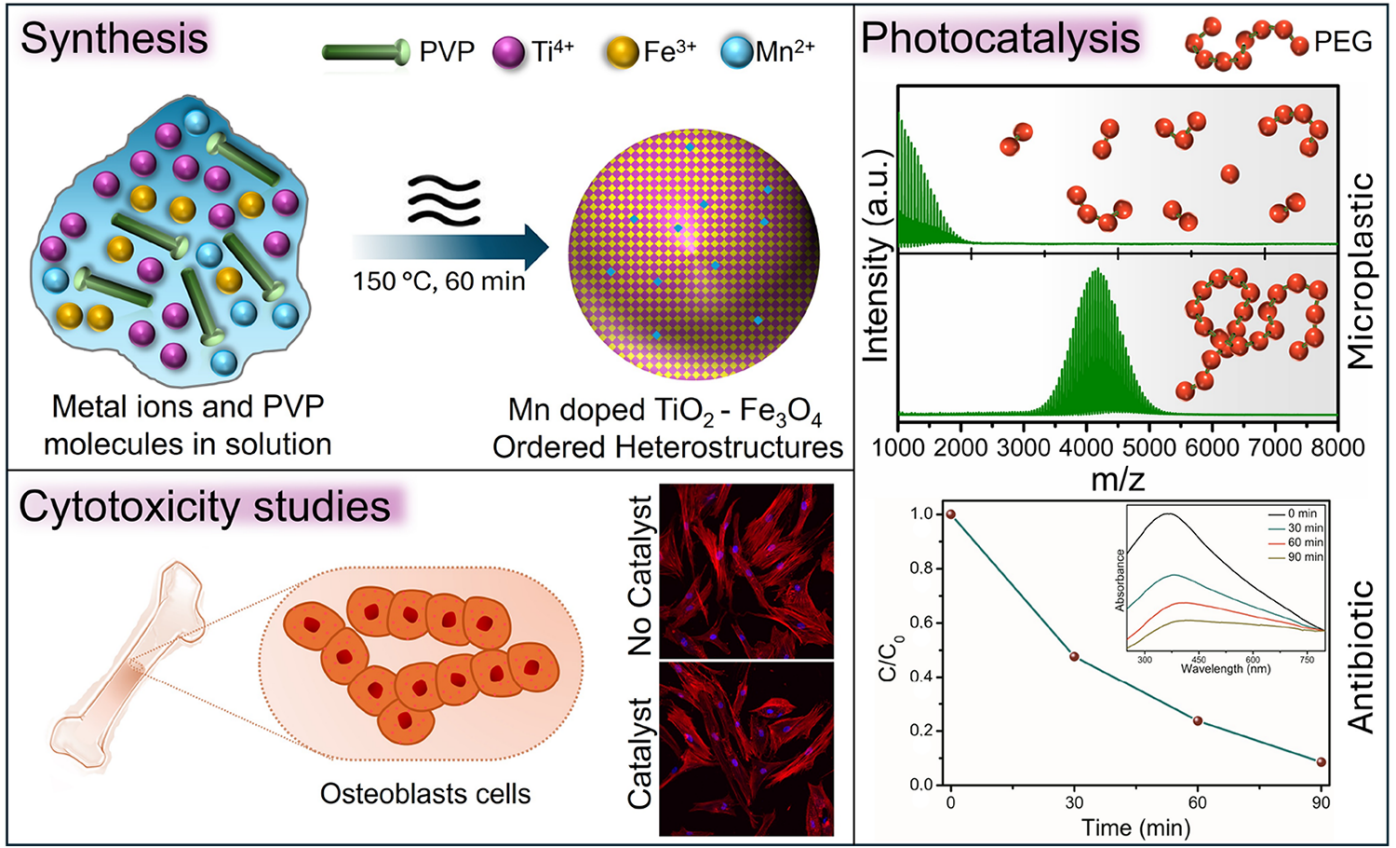
Schematic Illustration Outlining the Current Work where the Mn-Doped TiO_2_/Fe_3_O_4_ Ordered Heterostructures Prepared by Microwave Synthesis with Low Cytotoxicity Facilitate Photocatalytic Degradation of Microplastics and Antibiotics.

## 2 Results and Discussion

The affinity between metals and any ligands correlates with Lewis acidity and charge density, establishing a clear qualitative preference for the binding of various ions in solution. Specifically, Ti^4+^ is favored over Fe^3+^ and Mn^2+^ when binding to the carbonyl oxygen of polyvinylpyrrolidone (PVP).^30^ Titanium­(IV) is a highly hard, highly charged cation that effectively competes for oxygen donors. In contrast, Fe­(III) shares similar characteristics in terms of hardness and charge density, while divalent ions exhibit weaker binding affinities.^31^ The hierarchy of elements indicates that titanium and iron are mainly responsible for the initial complexation and nucleation processes in the mixed precursor solutions. In contrast, manganese plays a relatively minor role, typically appearing in only trace amounts, unless conditions are adjusted to enhance its incorporation. When subjected to microwave irradiation, the process involves rapid and uniform heating, resulting in higher local supersaturation levels that consequently accelerate the kinetics of nucleation.^32^ Consequently, this process results in the formation of numerous primary TiO_2_ and Fe_3_O_4_ nanocrystals within a brief period. After nucleation, the oriented attachment of primary nanocrystals by aligning lattice planes to minimize interfacial energy results in the formation of locally coherent nanodomains (Figure 1A). Ultimately, these aligned nanodomains come together to form micron-scale spherical heterostructures. X-ray diffraction (XRD), Fourier transform infrared (FTIR), and Raman spectra (Figure 1B–D) together confirm the formation of anatase-Fe_3_O_4_ heterostructures. However, no peaks corresponding to Mn were visible. A small amount of Mn is incorporated either by substituting into the Ti or Fe lattice sites or as extremely small MnO domains, which are below the detection limit of bulk probes but were detected using high-resolution transmission electron microscopy (HRTEM), energy-dispersive X-ray spectroscopy (EDS), and fast Fourier transform (FFT) patterns shown in Figure 2. Although the Mn signal is weak, its presence is confirmed locally in HRTEM and FFT, suggesting incorporation at trace levels sufficient to modify the electronic structure. Even at low atomic percentages of Mn, it can introduce defects or midgap states that alter the band alignment and improve charge separation, as has been shown for Mn-doped oxide systems.^33^ In general, the combined effects of PVP templating, affinity-driven precursor selectivity, microwave-accelerated nucleation, and oriented attachment result in a mesocrystal-like spherical heterostructure formation.

**Figure 1:**
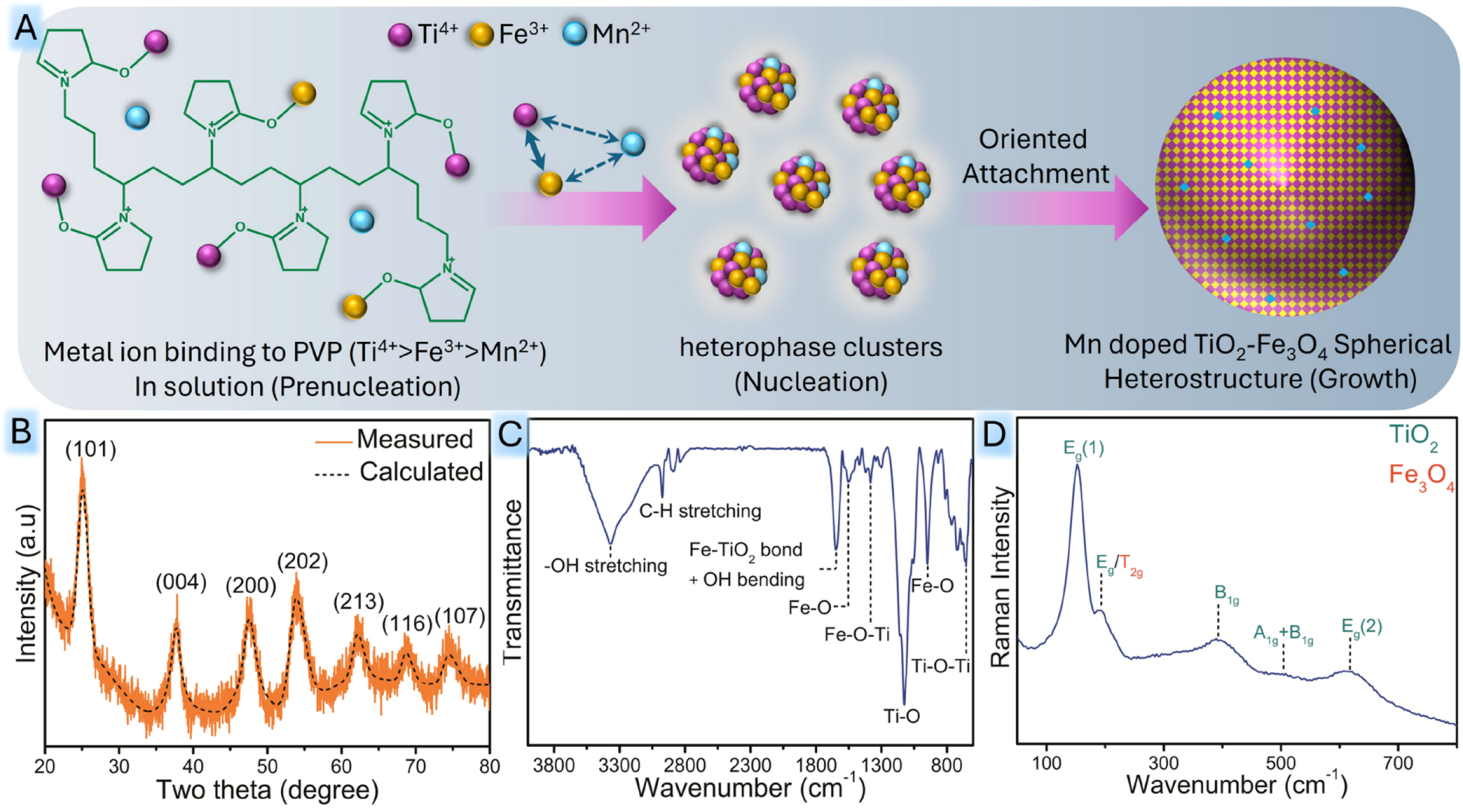
(A) Plausible mechanism for the formation of TFM. (B) XRD pattern of TFM. (C) FTIR spectrum of the heterostructure. (D) Raman spectrum of the heterostructure displaying characteristic vibrational modes.

**Figure 2:**
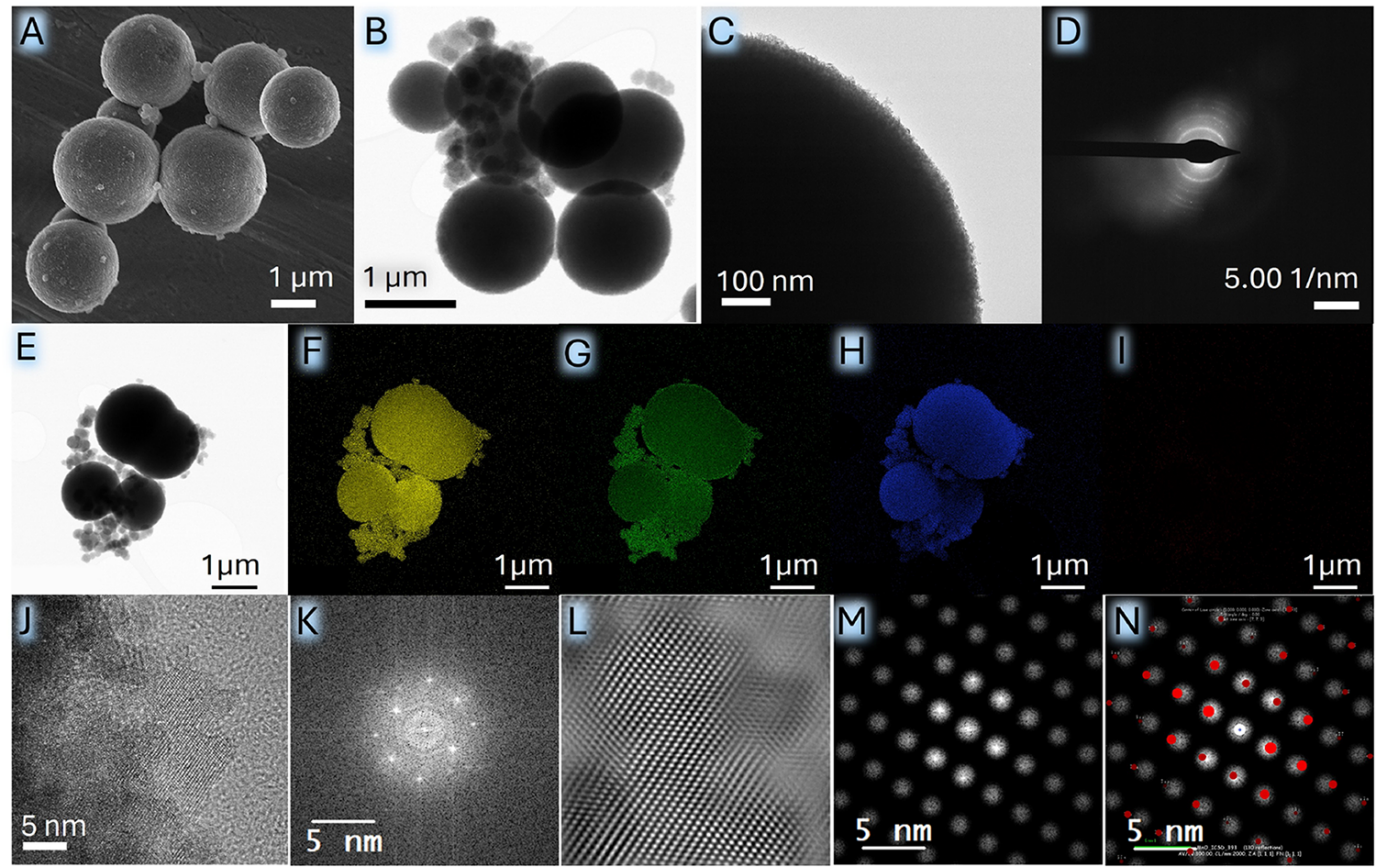
(A) Scanning electron microscopy (SEM) and (B) Transmission electron microscopy (TEM) images depicting the spherical morphology of TFM. (C) Enlarged TEM image depicting the ordering at the periphery of the heterostructure. (D) Selected area electron diffraction (SAED) pattern and (E) high-angle annular dark field-bright field (HAADF-BF) image showing the area under investigation and the corresponding elemental composition analysis of (F) Ti, (G) Fe, (H) O, and (I) Mn, (J) HRTEM image corresponding to MnO, and (K–N) corresponding FFT pattern.

The phase analysis and crystalline formation of TFM have been investigated by using powder XRD spectroscopy. The XRD spectrum of the synthesized heterostructure shown in Figure 1B has intense and sharp peaks, which indicate the good crystallinity of the sample. All the peaks in the spectrum correspond to the anatase phase of TiO_2_ (JCPDS No. 21-1272).^34^ Thus, the incorporation of the Fe component in the system will increase the anatase phase in the TiO_2_ crystal lattice, thereby increasing the overall photocatalytic property of TFM.^34^ The absence of the Fe_3_O_4_ phase confirms that the introduction of iron species did not alter the crystal phase of anatase TiO_2_ upon the addition of iron species to the TiO_2_ matrix; instead, it resulted in good crystallinity of the anatase phase of TiO_2_. The absence of the Mn peak in the spectrum can be due to the presence of a Mn dopant in trace amounts.

The XRD spectrum of the control sample synthesized without PVP shows anatase as the major phase (JCPDS No. 21-1272) along with the presence of a mixture of Fe_2_TiO_5_ (JCPDS No. 041-1432) and FeTi_2_O_5_ (JCPDS No. 089-8065) (Figure S1). Additionally, the peaks are broader and less intense, indicating lower crystallinity and a more amorphous nature for both phases. PVP acts as an efficient dispersant of the metal precursors and prevents agglomeration, controlling the size of the metal oxides.^35^ Furthermore, the presence of C–N, C=O, and CH_2_ functional groups in PVP, which enable TiO_2_ to act as a surface stabilizer, growth modifier, and nanoparticle dispersant, explains the presence of only the TiO_2_ phase in the XRD spectrum of TFM.^36^ In contrast, in the absence of PVP, the metal precursors are not well dispersed, which will result in aggregation of the metal oxides, as shown in the SEM images (Figure S2), and thus, Fe_3_O_4_ particles react with excess TiO_2_ to form Fe_2_TiO_5_ and FeTi_2_O_5_ phases as observed in the XRD spectrum.

The FTIR spectrum (Figure 1C) provides a clear insight into the active bonds and various functional groups present in TFM. The broadband at 3364 cm^–1^ represents the stretching vibration of the hydroxyl groups present in the surface-adsorbed water molecules, including the Ti–OH bond.^37^ The higher intensity of these bands signifies the presence of hydroxyl groups and water molecules in large amounts, which can increase the production of hydroxyl radicals during the photocatalytic process.^38^ Additionally, the band at 1648 cm^–1^ corresponds to the OH bending mode or can be assigned as vibration due to the Fe–TiO_2_ bond.^39,40^ Bands at 2975, 2890, and 2838 cm^–1^ are assigned to various C–H stretching vibrations, present due to the residual organic remains not being removed after washing.^40^ The bands at 1551 and 948 cm^–1^ can be attributed to the Fe–O bond.^41,42^ Several bands in the range of 600–800 cm^–1^ correspond to the vibrations of Ti–O–Ti bonds.^43^ The presence of a sharp band at 1126 cm^–1^ and a small band at 1383 cm^–1^ can be attributed to the stretching vibration of the Ti–O bond and the Fe–O–Ti bond, respectively, which hence gives a strong confirmation for the formation of TFM.^44^

The Raman spectra of TFM shown in Figure 1D exhibit six characteristic peaks located at 152, 192, 389, 507, and 612 cm^–1^, which correspond to the vibration modes of E_g_(1), E_g_, B_1g_, A_1g_ + B_1g_, and E_g_(2), respectively.^45^ The shoulder peak at 192 cm^–1^ can also be assigned as the T_2g_ vibrational mode of Fe_3_O_4_.^46^ The spectrum clearly indicates that TFM is mainly dominated by the anatase phase, which is consistent with the XRD results. The shift in peaks observed in the spectrum can be attributed to the incorporation of magnetite in sufficient proportion as the secondary component as well as the presence of Mn in the dopant amount.

The surface morphology and crystallinity of TFM were observed by using SEM and TEM (Figure 2). The SEM image shown in Figure 2A revealed a spherical morphology of TFM. This is further confirmed by the TEM measurements, which give a clear idea regarding the evolution of TFM. Figure 2B displays the TEM image of TFM with nanocrystals as substituents, and Figure 2C reveals the ordered alignment of the nanocrystals within the heterostructure. The TEM results depict the homogeneous substitution of Fe_3_O_4_ nanocrystals within the TiO_2_ matrix, which exhibit different phase compositions. The corresponding SAED pattern of the heterostructure in Figure 2D signifies the crystallinity of TiO_2_ and Fe_3_O_4_. The SAED pattern is composed of concentric rings, along with bright spots, and these rings correspond to the crystal planes of the anatase TiO_2_. Moreover, SAED patterns recorded at various locations appeared identical, indicating that the nanoparticles are uniformly distributed. HAADF-STEM and EDX analysis further confirm the composition of TFM. The HAADF-BF image shown in Figure 2E demonstrated a uniform distribution of Ti, Fe, and O (Figure 2F, 2G, and 2H, respectively) within the heterostructure. In contrast, Mn (Figure 2I) was found at a relatively low concentration. This is consistent with the elemental distribution revealed by the EDX mapping results. It is observed that the contents of Ti, Fe, and O elements in TFM are 34.9, 35.2, and 27.9 wt % (23.1, 19.9, and 55.4 atom %), respectively. EDX analysis detected only trace amounts of Mn; values below 1 wt % and atom % typically indicate that the elemental concentration is close to or below the detection limit of the method (Table 1 and Figure S3). However, certain FFT patterns could potentially correspond to the MnO phase. Figure 2J shows the HRTEM lattice image indicating MnO, and the corresponding FFT patterns are presented in Figure 2K–N. The HRTEM lattice images confirming the anatase and Fe_3_O_4_ phase are shown in Figure S4, where the marked lattice spacing of 0.35 nm corresponds to the (101) plane of anatase TiO_2_,^47^ and the lattice fringe of 0.50 nm is attributed to Fe_3_O_4_.^48^

**Table 1:** Ti, Fe, Mn, and O Elemental Distribution in TFM Obtained from TEM-EDX

	Ti	Fe	Mn	O
atom %	23.1	19.9	0.3	55.4
wt %	34.9	35.2	0.5	27.9

The existence of defects in TFM system was investigated by conducting photoluminescence (PL) studies (Figure 3A). The intensity of the peaks appearing in the PL helps identify various defect states present in TFM and is closely related to their photocatalytic efficiency. This analysis provides a clear picture of the recombination behavior and charge carrier transport of the photocatalytically generated electron–hole pairs. The presence of iron oxide as a secondary component and manganese ions in the dopant amount in the TiO_2_ matrix generates intrinsic defects, which create deeper energy levels within the band gap, thereby extending light absorption and increasing photocatalytic efficiency.^49^ The PL spectra of TFM (Figure 3A), excited at 325 nm, identify 12 significant peaks at 400, 413, 426, 442, 453, 462, 468, 482, 491, 515, 528, and 537 nm. The position of these peaks in the visible range can be attributed to the presence of defects, oxygen vacancies, and surface states in the heterostructure. The peaks within the range of 400–450 nm correspond to the emission peaks of the self-trapped excitons.^50^ The PL peaks between 450 and 510 nm can be assigned as emission peaks from the trap states within the band gap due to oxygen vacancies and defects and the presence of other surface states. The emission peaks located around 515, 528, and 537 nm can be due to the presence of oxygen vacancies within the bulk of the heterostructure. It is evident from the spectra that the emission peaks resulting from surface oxygen vacancies and defects are the major peaks, whereas the peaks originating from oxygen vacancies inside the heterostructure were less intense. Thus, it can be inferred that the majority of the defects were present on the surface.^50^

**Figure 3:**
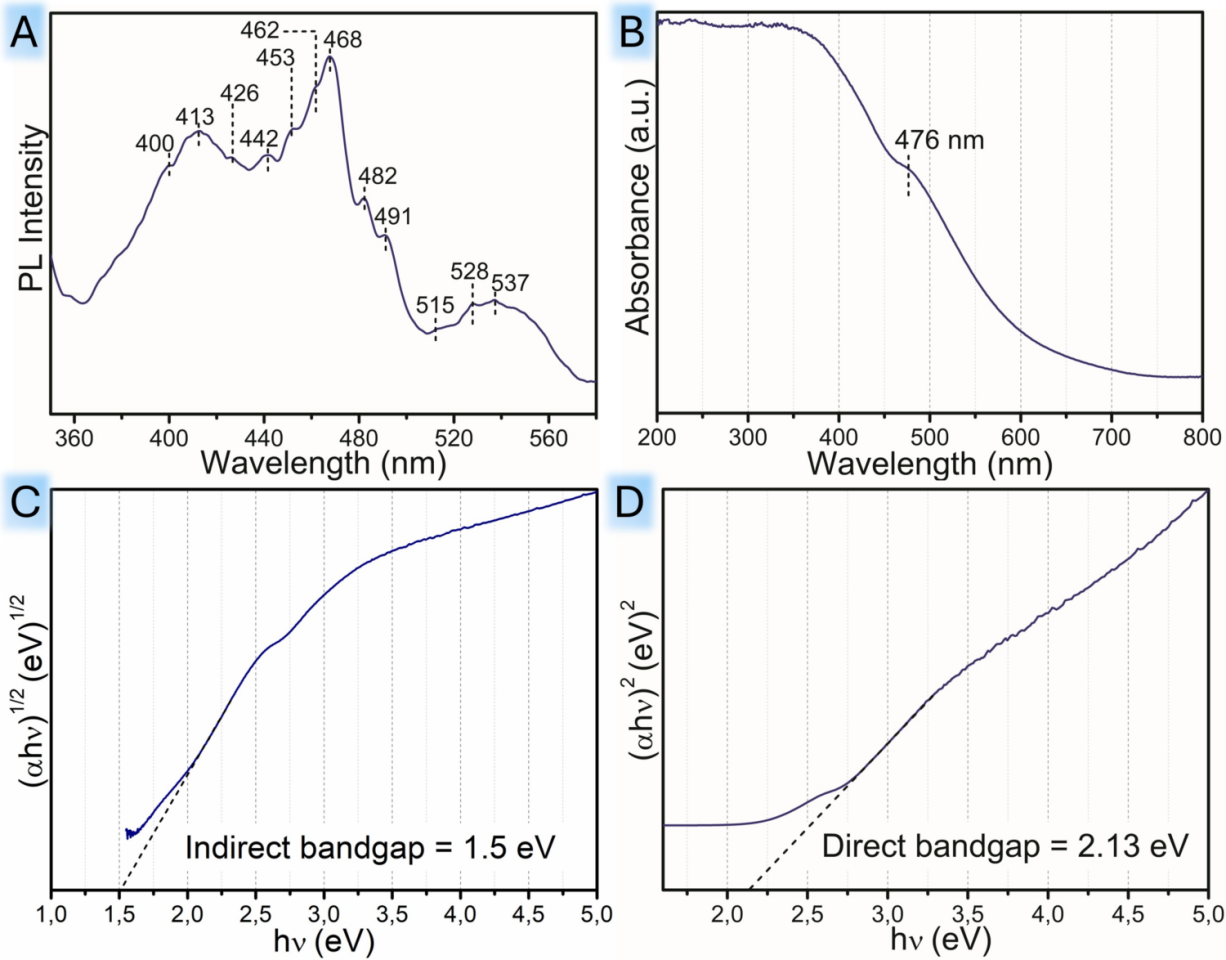
(A) PL spectra of TFM recorded at an excitation wavelength of 325 nm. (B) UV–visible absorption spectrum showing the absorption range and the peaks corresponding to the d–d transition due to the interaction of iron species with TiO_2_. The Tauc plot of TFM for (C) *n* = 1/2 and (D) *n* = 2.

The defect richness in TFM also improves the optical properties. Figure 3B–D shows the UV–visible absorption spectra and s Figure 3C and D show the Tauc plots of of TFM. From the spectra, it is observed that the TFM system has significantly higher absorption between 400 and 700 nm. The introduction of iron species and the Mn dopant to the TiO_2_ matrix resulted in a red shift of the absorption edge to the visible region, resulting in the dark brown color of TFM.^51^ The enhancement in absorption can be assigned to the charge transfer transition of the interacting iron ion species with the TiO_2_ band gap.^52^ The absorption band at λ = 476 nm can be attributed to the d–d transitions between T_2g_ → A_2g_ and T_2g_ → T_1g_ of the Fe^3+^ ion or the Fe^2+^ + Fe^3+^ → Fe^3+^ + Fe^2+^ charge transfer transition.^53^ The formation of structural disorders upon the addition of Fe species in the crystal lattice introduces midgap states between the conduction and valence band edges, ultimately leading to band gap narrowing. From the Tauc plot shown in Figure 3C, the band gap energy for the composite was found to be 1.5 eV. This optical band gap value was obtained by plotting (α*h*ν)^1/2^ vs (*h*ν). The linear portion of the graph was obtained when *n* = 1/2, which corresponds to an indirect allowed transition between the conduction band and valence band. The influence of the Mn dopant is also crucial in achieving such an optical band gap, alongside the contribution of Fe_3_O_4_.^10^ Figure 3D shows the Tauc plot corresponding to direct allowed transitions (*n* = 2), and the optical band gap in this case is 2.13 eV.

The chemical state of the surface Ti, Fe, and O elements in TFM was analyzed by XPS (Figure 4). The elemental composition on the surface of TFM, analyzed using a wide survey XPS spectrum in Figure 4A, exhibits peaks representing Ti 2p, O 1s, and Fe 2p, with no peaks corresponding to Mn 2p, which confirms that the MnO concentration is beyond the XPS detection limit.^54^ For the Ti 2p spectrum shown in Figure 4B, two major peaks at 459.9 and 465.4 eV can be ascribed to Ti 2p_3/2_ and Ti 2p_1/2_, respectively, which indicate the presence of the Ti^4+^ state in the crystal lattice. Additionally, the lower binding energy shoulder peaks at 457.5 and 463.2 eV correspond to the Ti^3+^ point defect state, signifying the presence of surface oxygen vacancies.^55^ For the Fe 2p spectrum in Figure 4C, a total of six peaks were observed, with the major peaks located at 711.4 and 725.2 eV corresponding to the Fe 2p_3/2_ and Fe 2p_1/2_ states of Fe^2+^ in Fe_3_O_4_, respectively.^56^ The other two distinct peaks at 713.4 and 727.4 eV are due to 2p_3/2_ and 2p_1/2_ of the Fe^3+^ state, respectively. The remaining peaks positioned at 720.5 and 734.1 eV are the satellite peaks, indicating the formation of the Fe_3_O_4_ phase.^56^ The comprehensive performance of both Fe^2+^ and Fe^3+^ ions in the Fe_3_O_4_ phase resulted in a greater number of peaks in the Fe 2p spectrum. The O 1s spectra in Figure 4D are deconvoluted to four peaks positioned at binding energies of 528.4, 529.8, 531.5, and 533.2 eV. The peak at 528.4 eV arises due to O^2–^ ions present in TFM lattice. The peak observed at 529.8 corresponds to the O^2–^ deficiency within the heterostructure or as Ti^3+^–O/Ti^4+^–O.^57^ The peaks at 531.5 and 533.2 eV are possibly due to loosely bound oxygen atoms on the surface and Ti–OH, respectively. These results strongly support that the introduction of Fe species induces a higher number of oxygen vacancies and Ti^3+^ defects in TFM. Also, in the O 1s spectra, a shift of peak values is observed in TFM compared to pristine TiO_2_, corresponding to a strong interaction between Fe_3_O_4_ and TiO_2_.^58^ The valence band XPS spectra shown in Figure 4E exhibit a prominent shift from 0.36 to −0.56 eV due to significant band tailing near the valence band edge. The valence band density of states (DoSs) shown in Figure 4F,4G provides clear evidence of defects, surface oxygen vacancies, and Ti^3+^ centers in the crystal lattice. These intrinsic defects lead to significant alterations in the electronic structure of the heterostructure by modifying the conduction band and valence band positions and introducing localized midgap states near the top/bottom of their valence/conduction band.^59^ The upward shift of the valence band edge (0.92 eV) is indicative of a higher density of defect levels due to surface oxygen vacancies. Additionally, the existence of Ti^3+^ defects creates shallow donor states below the conduction band edge, leading to a slight CB tailing.^60^ The conduction band minimum would occur at 1.5 eV higher than at −2.06 eV, as shown in the DoS profile in the case of indirect allowed transition (Figure 4F). For the directly allowed transition, the conduction band minimum would occur at 2.13 eV higher than at −2.69 eV, as shown in Figure 4G. Thus, the combined effect of oxygen vacancies, Ti^3+^ defects, and Fe ions in the ordered heterostructure introduces active midgap defect states and an edge tailing phenomenon, leading to remarkable band gap narrowing and enhanced light absorption, which is beneficial for photocatalysis.

**Figure 4:**
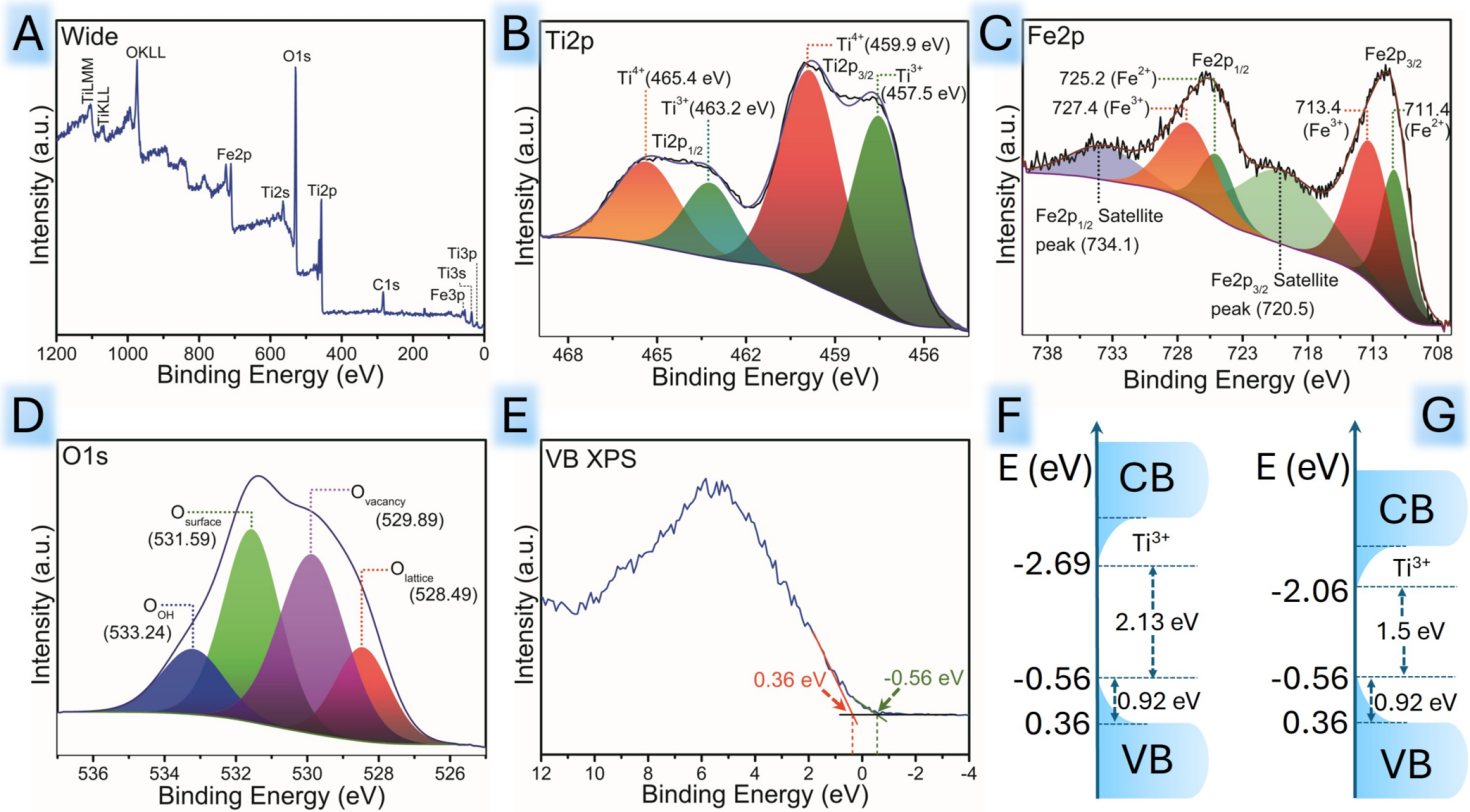
XPS spectra of TFM showing the oxidation states and splitting of metal and nonmetal ions, (A) wide spectrum, (B) Ti 2p, (C) Fe 2p, (D) O 1s, (E) valence band (VB) XPS spectrum depicting the VB tailing and narrowing of band gap energy for TFM, and (F, G) corresponding density of states showing the energy levels corresponding to direct and indirect band gap transitions.

The XPS data of the control sample also exhibit a similar pattern and the presence of peaks corresponding to Ti 2p, O 1s, and Fe 2p and no peaks representing Mn 2p (Figure S5). For the Ti 2p spectrum, the peaks at 458.7 and 464.5 eV are attributed to Ti 2p_3/2_ and Ti 2p_1/2_, respectively, indicating the presence of the Ti^4+^ state, and the shoulder peak at 456.8 corresponds to the Ti^3+^ point defect state in the lattice.^55^ The Fe 2p spectrum identifies peaks corresponding to the Fe^2+^ state (711.2 and 724.7 eV) and the Fe^3+^ state (713 and 726.6 eV) from the mixed metal oxide phases. The remaining peaks with binding energies at 720 and 733.8 eV are satellite peaks.^56^ For the O 1s spectrum, the peaks at 530.1 and 530.6 eV correspond to the lattice oxygen and the presence of oxygen vacancies in the crystal lattice, respectively, and the peaks at 531.7 and 533.7 eV are attributed to loosely bound oxygen atoms and oxygen in the hydroxyl group on the surface, respectively.^56,57^

### 2.1 Cytotoxicity Studies

#### 2.1.1 Cell Viability

The results of the (3-(4,5-dimethylthiazol-2-yl)-2,5-diphenyltetrazolium bromide) (MTT) assay performed upon either 24 or 72 h treatment with four different concentrations of TFM suspensions are illustrated in Figure 5 and Table 2. According to the ISO 10993-5:2009 standard, the threshold for cytotoxicity is 70% cell viability (denoted in Figure 5 as a red dotted line), meaning that values below 70% indicate a cytotoxic effect (grade 1–4), while values ≥70% are considered noncytotoxic (grade 0).

**Figure 5:**
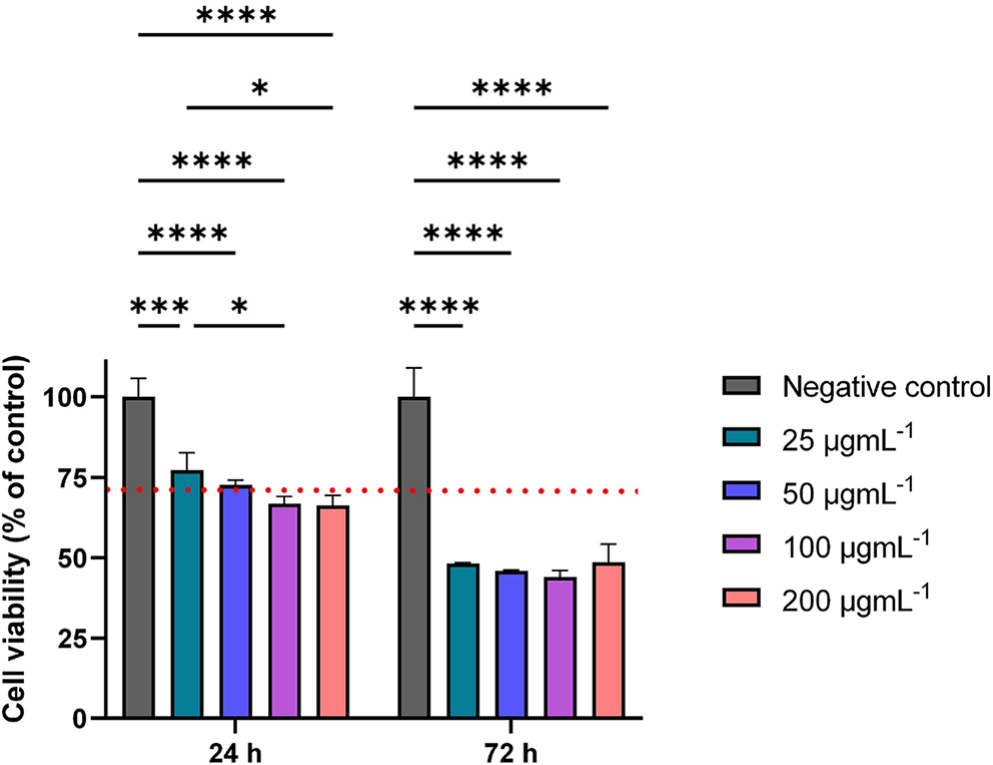
Results of the cell viability assay carried out for osteoblasts exposed to TFM for 24 and 72 h. Data are presented as mean ± standard deviation. The negative control refers to cells which were not exposed to heterostructure suspensions. Asterisks (****) denote statistical significance (*p* < 0.0001).

**Table 2:** Percentage Osteoblast Cell Viability Values Were Calculated as Absorbance-Corrected with the Background Signal and Referenced to the Negative Control

	negative control	25 μg mL^–1^	50 μg mL^–1^	100 μg mL^–1^	200 μg mL^–1^
24 h	100 ± 4.7	77.3 ± 4.4	72.6 ± 1.2	66.8 ± 1.8	66.3 ± 2.5
72 h	100 ± 7.4	48.2 ± 0.3	45.9 ± 0.3	44 ± 1.6	48.6 ± 4.6

Overall, a few consistent patterns may be observed in the viability of cells exposed to TFM. The differences in osteoblast viability between the negative control and all tested concentrations were always statistically significant, indicating that the heterostructure had a measurable impact on the cell viability. Moreover, prolonged exposure to TFM led to a further drop in cell viability, suggesting an increased cytotoxicity over time. Upon 24 h treatment, the heterostructure induced either a noncytotoxic response (up to 50 μg mL^–1^) or slight cytotoxicity, whereas 72 h exposure resulted in mild cytotoxicity in osteoblasts regardless of the concentration studied. The lack of a clear dose–response relationship, especially with extended exposure, implies that the cytotoxic effects may not be driven only by the concentration of TFM or its physicochemical properties but could also involve other factors, such as agglomeration tendency, cell uptake, reactive oxygen species formation (a well-documented phenomenon for iron and its compounds), or alterations in cellular metabolism.

#### 2.1.2 Cell Morphology

The representative confocal laser scanning microscopy (CLSM) images, depicted in Figure 6, showcase the effects of TFM on osteoblasts following either 24 or 72 h of incubation. It may be observed that all cells exposed to the investigated heterostructure for 24 h exhibited a phenotype similar to the control group. Flattened and polygonal shapes were prevailing among osteoblasts, although some of them adopted a fusiform (spindle-like) morphology as well. The formation of cytoplasmic extensions and an even distribution of cells in the imaged area are also worth mentioning. In fact, no apparent discrepancies in the morphology and behavior of osteoblasts could be distinguished between any of the heterostructure-treated cells upon 24 h exposure, which coincides well with the MTT assay data pointing to noncytotoxicity or slight cytotoxicity. On the other hand, osteoblasts treated for 72 h clearly differed from the heterostructure-free control group. A lower number of cells in the imaged area, with a vast number of them exhibiting an elongated morphology, was easily noticeable. Some processes observed upon prolonged incubation of osteoblasts with TFM included cell blebbing (the formation of rounded cells), cytoskeleton depolymerization (breakdown of actin filaments, further resulting in cell shrinkage), and nuclear condensation (in fact, chromatin condensation yielding a clearly visible, smaller nucleus). These are typically cell death-related processes, indicating that the prolonged exposure to TFM induced cytotoxicity in osteoblasts (as already indicated by the MTT assay).

**Figure 6:**
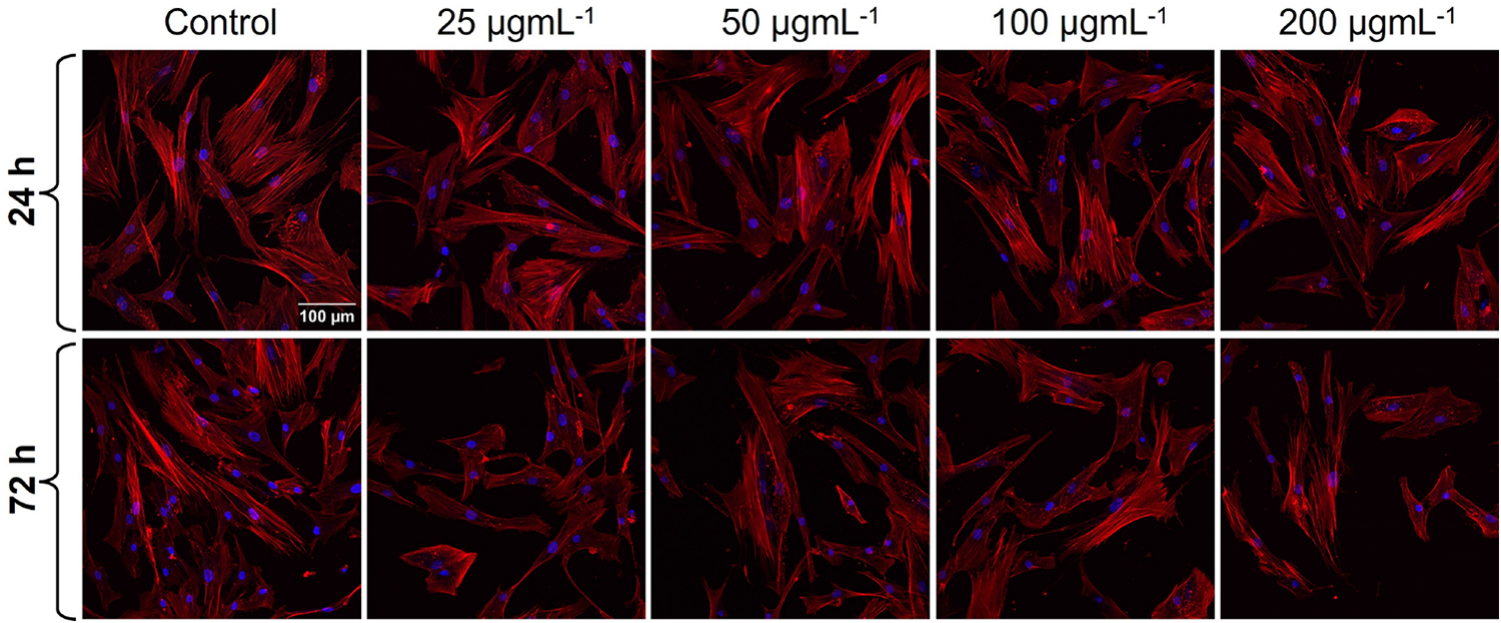
Morphologies of osteoblasts exposed to differently concentrated TFM for 24 and 72 h. The F-actin of the cytoskeleton and cell nuclei were stained with Rhodamine Phalloidin (red color) and Hoechst 33342 (blue color), respectively.

### 2.2 Photocatalysis

#### 2.2.1 Photodegradation of Microplastics (PEG)

In this work, TFM was used for the capture and photocatalytic degradation of polyethylene glycol (PEG-4000). PEG 4000 was used since it has a high molecular weight, and the resulting fragments can be easily detected by matrix-assisted laser desorption/ionization-time-of-flight (MALDI-TOF).^26^ PEG samples with a heterostructure in the presence and absence of H_2_O_2_, PEG samples with H_2_O_2_, and PEG samples alone were subjected to UV irradiation for 24 h. Figure 7A depicts the MALDI-mass spectrometry (MALDI-MS) spectra of PEG, UV-irradiated PEG, PEG with H_2_O_2_, and PEG with the heterostructure in the presence and absence of H_2_O_2_. Signals corresponding to PEG macromolecular chains at around *m*/*z* values of 4000 stay intact after exposure to 24 h UV irradiation (Figure 7A). The spectra of PEG samples with H_2_O_2_ show a decrease in signal intensity around *m*/*z* 4000, and new peaks at lower mass regions are generated with the highest mass intensities (Figure 7A). This result is in agreement with the previous studies of the PEG photodegradation mechanism, wherein H_2_O_2_ is decomposed to form OH radicals upon UV irradiation, and these radicals attack the C–O bonds of PEG, producing oxidized polymer chains at lower mass regions.^26^ The MALDI spectra of the control experiment with a bare heterostructure show that the intensity of peaks in the higher mass region is decreased to some extent (Figure 7A). The MALDI spectra of PEG samples with the heterostructure and H_2_O_2_ exhibit a high rate of fragmentation with increased intensity of oxidized fragments below *m*/*z* values of 1000 (Figure 7A). These results reveal the superior PEG degradation ability of heterostructures in the presence of H_2_O_2_, which is attributed to their enhanced interfacial charge transfer, improved charge separation, and ability to produce more ROS species during photocatalysis. Hence, the synergism between H_2_O_2_ and the catalyst has been revealed. PEG degradation involves two main reactions: photocatalysis and photo-Fenton reaction. The control experiments conducted in the presence of H_2_O_2_ and its absence involve the sole photo-Fenton reaction and photocatalysis, respectively, leading to poor PEG degradation in the former case and negligible degradation in the latter. The excellent PEG degradation depicted in the MALDI result in Figure 7A is attributed to the synergistic reactions of the photocatalytic reaction and the photo-Fenton process.

**Figure 7:**
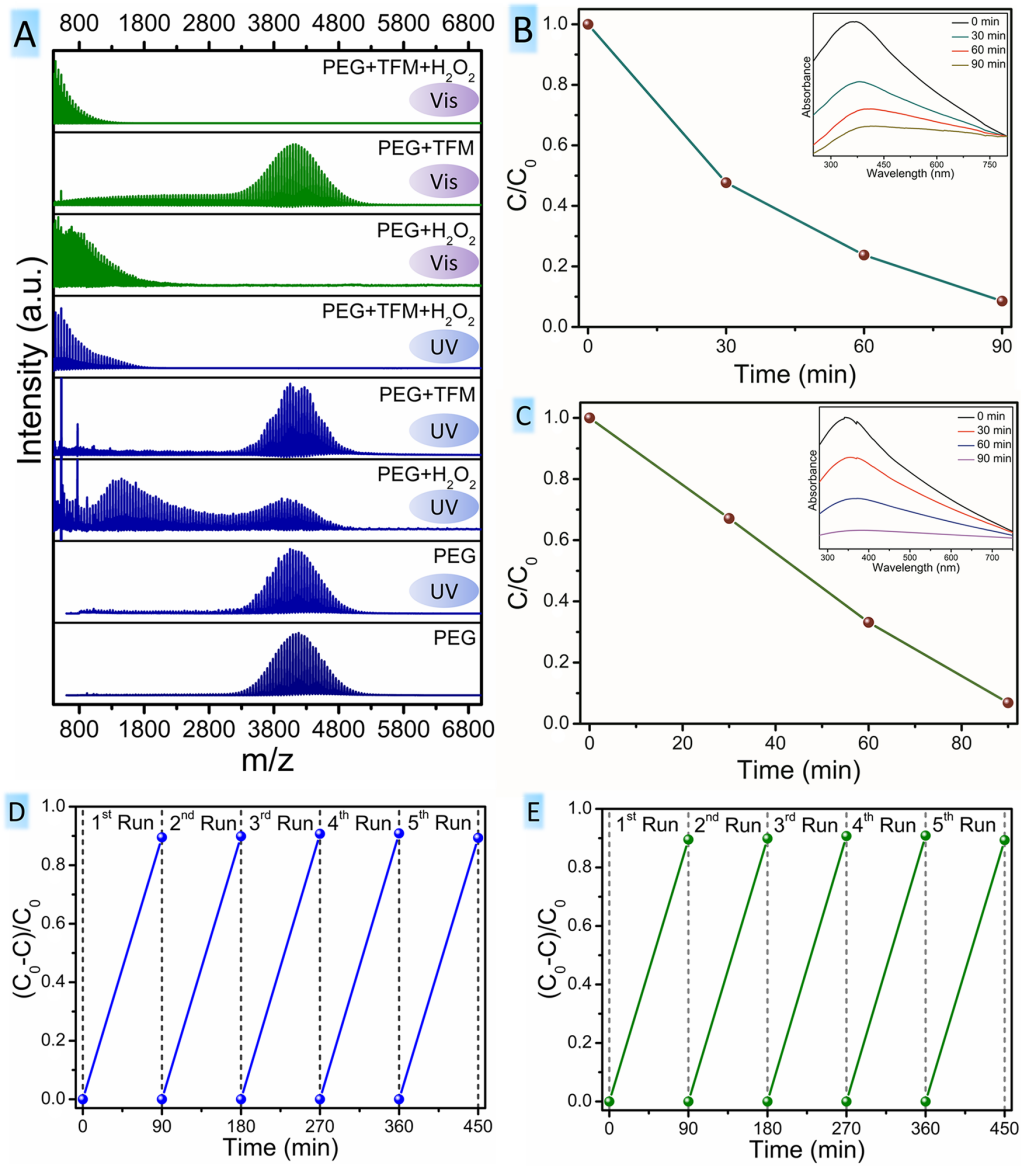
(A) MALDI results of PEG fragmentation using PEG alone, PEG after 24 h of UV irradiation, PEG + H_2_O_2_ after 24 h of UV irradiation, PEG + TFM after UV irradiation, PEG + TFM + H_2_O_2_ after 24 h of UV irradiation, PEG + H_2_O_2_ after 24 h of visible light irradiation, PEG + TFM after visible light irradiation, and PEG + TFM + H_2_O_2_ after 24 h of visible light irradiation. (B) Photodegradation efficiency of tetracycline (inset showing the UV–vis absorption spectrum of photodegradation) under UV irradiation. (C) Photodegradation efficiency of tetracycline (inset showing the UV–vis absorption spectrum of photodegradation) under visible light irradiation. (D) Reusability experiments for the TC degradation over five repeated cycles under UV irradiation. (E) Reusability experiments for the TC degradation over five repeated cycles under visible light irradiation. (*C*_0_ is the initial absorbance and *C* is the absorbance at a specific time for TC degradation).

The photocatalytic activity of TiO_2_ is a well-known mechanism. Ullattil et al. have demonstrated the ability of TiO_2_ as a single-component asymmetric superstructure microrobot for the phototrapping and photodegradation of microplastics.^61^ Upon photoexcitation, the TiO_2_ catalyst generates electron–hole pairs (eq 1). The holes, when reacted with surrounding water molecules or with hydrogen peroxide present in the medium, generate hydroxyl radicals (eq 2). In contrast, the electrons, in reaction with adsorbed oxygen molecules, generate superoxide radicals (eq 3).

1 $${\mathrm{TiO}}_{2}+h\nu \to {\mathrm{TiO}}_{2}\;\left(h^{+}+e^{-}\right)$$

2 h++H2O→H++OH•

3 $$O_{2}+e^{-}\to O_{2}^{•-}$$

Mn modification in the heterostructure would effectively suppress the charge carrier recombination by effectively separating the photogenerated electron–hole pairs. The enhanced photocatalytic properties of the heterostructure for PEG degradation can be attributed to the presence of Fe_3_O_4_. Iron oxides, when present in the medium containing H_2_O_2_, initiate the Fenton reaction upon UV irradiation.

The photo-Fenton mechanism is demonstrated by the following equations^28^

4 $${\mathrm{Fe}}_{3}O_{4}+h\nu \to {\mathrm{Fe}}_{3}O_{4}\;\left(h^{+}+e^{-}\right)$$

5 H2O2+e−→OH−+OH•

or

6 $${\mathrm{Fe}}^{3+}+e^{-}\to {\mathrm{Fe}}^{2+}$$

7 Fe2++H2O2→Fe3++OH−+OH•

Fe_3_O_4_ generates electron–hole pairs under light illumination (eq 4). The electrons are then trapped by H_2_O_2_, creating hydroxyl radicals (eq 5), or by reducing Fe^3+^ to Fe^2+^ (eq 6), which then react with H_2_O_2_ to generate hydroxyl radicals (eq 7).

As evidence, Fe_2_O_3_ (hematite) has shown a high rate of photofragmentation of PEG under UV irradiation.^26^ This advanced oxidation process accelerates the production of hydroxyl radicals in the system. The interconversion of Fe^3+^ and Fe^2+^ in our photocatalytic system is supposed to catalyze the generation of hydroxyl radicals. Thus, Fe^3+^/Fe^2+^ redox coupling increases the production of hydroxyl radicals, which play a dominant role in the photofragmentation and facilitate the chain scission process to yield oligomers and lower molecular mass oxidized fragments of PEG.

Furthermore, the catalyst exhibits a higher fragmentation rate under visible light irradiation as shown in Figure 7A, which strongly supports the visible wavelength absorption of TFM observed in the UV–vis spectrum. Compared to UV irradiation, visible light resulted in better activity for PEG samples with H_2_O_2_ and with TFM. The MALDI spectra of PEG samples with TFM and H_2_O_2_ further enhanced the fragmentation, which can be attributed to the synergism between the catalyst and hydrogen peroxide under visible light.

PEG-4000 when exposed to both UV and visible light is fragmented to oligomers with hydroxyl, aldehydic, and formic acid end groups.^62^ A table summarizing the literature depicting PEG degradation to date is shown in Table S1, and another table showing the possible PEG degradation fragments with their actual and calculated molecular weights below 1000 Da is summarized in Table S2. The minor difference between the actual and calculated molecular weights of the oligomers can be attributed to the instrument parameters and sample preparation factors, including the choice of solvent, reagent, and type of matrix, all of which will influence the *m*/*z* value.^63,64^

#### 2.2.2 Photodegradation of Tetracycline

The photocatalytic potential of TFM was evaluated using a model degradation study involving tetracycline (TC). Before irradiation, TFM catalyst, along with TC, was kept in the dark for 2 h to eliminate any initial adsorption effect. Upon illumination, TC started to degrade, evident from Figure 7B. The inset image shows the UV–vis absorption spectrum of TC degradation at different time intervals (inset, Figure 7B). It has been found that TC undergoes nearly complete degradation within 90 min of UV irradiation. A comparative table summarizing the key literature reports of TC degradation under similar conditions is shown in Table S3.

TFM enhances the quantum efficiency during the photocatalytic process by suppressing charge carrier recombination. The ordered characteristic feature of TFM promotes anisotropic electron flow and improved photoactive efficiency due to enhanced charge separation or facile charge separation. The antibiotic degradation exhibited by TFM is attributed to the synergistic effect of Fe_3_O_4_, TiO_2_, and Mn^2+^ ions. Fe_3_O_4_ plays a crucial role in electron transfer and conversion as a functional center.^7^ The incorporation of Fe_3_O_4_ in TFM apparently results in band gap narrowing due to the intrinsic defects formed, as evident from the PL spectrum. These intermediate states suppress the recombination of photogenerated electron–hole pairs by interfacial electrons, thus increasing the lifetime of the charge carriers. The Mn ions present in the system would also help in the modification of the crystal structure and suppress the recombination of photogenerated charge carriers, thereby increasing their lifetime. The presence of Mn as MnO, as indicated by the TEM analysis, promotes the generation of hydroxyl and superoxide radicals.^9,10^ When radiated with a UV light of 365 nm, TFM effectively participates in the TC degradation process. The photodegradation is initiated by the generation of an electron–hole pair by TiO_2_.^65^ The enhanced absorption of the photocatalyst, as evidenced from the UV–visible spectrum, resulted in the effective utilization of the UV light, and thus, the valence band electrons are easily excited, while the photogenerated holes undergo an oxidation process with the water molecule or hydroxide ions, generating hydroxyl radicals. Fe_3_O_4_ potentially acts as an electron sink, where the electrons are trapped by the Fe_3_O_4_-induced trap intermediate state, enabling the reduction of Fe^3+^ to Fe^2+^ and favoring e–h separation. The electrons thus migrated to reach the catalytic surface of TFM and reduce the adsorbed oxygen to form superoxide radicals. Additionally, the Fe^3+^/Fe^2+^ redox cycle facilitates the production of superoxide radicals in the system, accelerating the degradation.^8^ Therefore, the photochemically generated holes and hydroxyl and superoxide radicals facilitate the photodegradation of TC to nonhazardous products.

In a different context, when the catalyst is introduced in more challenging matrices such as in industrial wastewater containing high concentrations of halides and carbonate ions, this can promote the production of versatile radical species for pollutant degradation. Halide ions and carbonate ions act as OH radical scavengers, forming reactive halogen species (RHS) and carbonate radicals, respectively, which can increase the preference toward electron-rich moieties such as aromatic rings and NH_2_ and OH groups in the TC structure.^66^

During the photodegradation of TC, the UV–vis absorption spectrum has shown a red shift for the TC peaks from 363 to 395 nm (inset of Figure 7B). The TC degradation follows first-order kinetics, and the first-order linearization curve is obtained by plotting ln­(*C*_0_/*C*) vs time, as shown in (Figure S6). Notably, the catalyst exhibited improved degradation efficiency under visible light irradiation, after the same irradiation time, compared to that under UV irradiation (Figure 7C).

#### 2.2.3 Reusability of TFM Photocatalyst

To investigate the catalyst reusability, a cycle experiment of TC degradation was conducted with both UV and visible light irradiation. It was found that TFM remained photocatalytically active in multiple utilization cycles, which demonstrates the efficiency and stability of the catalyst for repeated usage (Figure 7D,E).

#### 2.2.4 Scavenger Experiments and Proposed Mechanism

To clarify which reactive species contribute to the photocatalytic degradation of TC, scavenging experiments were performed using isopropanol (IP) as a ^•^OH scavenger, hydroquinone (HQ) as a ^•^O_2_^–^ scavenger, and ethylenediaminetetraacetic acid (EDTA) as a h^+^ scavenger.^67^ The absorption spectrum of TC after dark adsorption showed a broad peak with a maximum intensity at 363 nm (0.7 au), resulting from a combination of π–π* (∼265 nm) and n−π* (∼365 nm) transitions of its aromatic and amide groups.^68^ After 90 min of UV exposure without scavengers, this band shifted to 395 nm with the intensity dropped to 0.04 au, indicating near-total mineralization of TC. From Figure S7, it is clear that with IP, only a peak at 265 nm (0.19 au) remained, suggesting that ^•^OH radicals have a minor role. Conversely, HQ and EDTA caused significant increases in absorbance (3.9 and 4.7 au, respectively), accompanied by a loss of absorption above 315 nm (HQ) and 415 nm (EDTA). This implies that the aromatic chromophores are broken down, but many low-molecular weight fragments form, confirming that both ^•^O_2_^–^ and h^+^ species are involved in the degradation. The strong inhibition by EDTA indicates that photogenerated holes are the main oxidants, driving direct electron removal from TC. The degradation pathway involves h^+^-induced oxidation followed by ^•^O_2_^–^-mediated breakdown, with ^•^OH radicals playing a minor role. We acknowledge that direct ROS detection would further support our mechanistic conclusions, and these operando spectroscopic studies are in progress and will be addressed in future research.

## 3 Conclusions

A one-pot microwave synthetic strategy for synthesizing Mn-doped TiO_2_–Fe_3_O_4_ ordered heterostructures has been developed. The resultant heterostructures, featuring crystallographically aligned nanocrystals, exhibit enhanced functionality and excellent properties due to the synergistic effects between TiO_2_ and Fe_3_O_4_, combined with trace doping of Mn, resulting in improved photocatalytic degradation efficiency toward ubiquitous contaminants. The detailed characterizations of the material provide valuable insights into both its composition and ordering, highlighting its structural features. Prior to photocatalysis, the cytotoxic evaluation has been conducted with osteoblast cells, which demonstrated that the viability of osteoblasts exposed to TFM was above the ISO 10993-5:2009 cytotoxicity threshold (≥70%) at concentrations of up to 50 μg mL^–1^ after 24 h, whereas higher doses (100–200 μg mL^–1^) and extended exposure (72 h) elicited a mild but statistically significant drop in viability (down to ∼45–50% of the untreated control group). Interestingly, the absence of a strict dose–response relationship, specifically upon 72 h treatment, and a generally favorable role Mn brings about on cells suggests that there are factors beyond concentration, such as nanoparticle agglomeration, cellular uptake dynamics, or Fe-mediated ROS generation, that could influence cytotoxic outcomes. The photofragmentation of a skeptical microplastic, PEG, and a widely used antibiotic, tetracycline, has been used as model pollutant systems to evaluate the photocatalytic efficiency of the newly developed heterostructure. As a result, the successful photofragmentation of PEG and photodegradation of tetracycline have been observed with evidence of the prominent roles of holes and superoxide radicals, along with marginal effects of hydroxyl radicals. Hence, this article opens a pathway toward multicomponent, ordered heterostructures for the photocatalytic degradation of pollutants that pose a threat to ecosystems and human health, taking into account that their cytocompatibility is limited to short-term exposure and lower concentration regimes.

## 4 Experimental Section

### 4.1 Synthesis of TFM

Synthesis: 0.253 g of polyvinylpyrrolidone (PVP, average *M*_w_ = 40,000) was dissolved in 100 mL of deionized (DI) water containing 6 mmol TiOSO_4_. After 10 min of stirring, 50 mL aqueous solutions of 3 mmol FeCl_3_·6H_2_O (Merck, >97%) and MnSO_4_·H_2_O (Merck, ≥99%) were added to the mixture separately. Hence, the ratio of metal (Ti/Fe/Mn) ions is kept at 2:1:1. Stirring was continued for another 15 min with microwave irradiation at 150 °C for 60 min at a set microwave power of 850 W. The reaction mixture was then cooled to 60 °C. The obtained sample was then washed with DI water and absolute ethanol (Penta chemicals) followed by vacuum drying at 60 °C for 90 min.

### 4.2 Cytotoxicity Studies

Cytotoxicity of TFM was investigated through cell viability investigations based on an MTT assay as well as cell morphology analysis via confocal laser scanning microscopy (CLSM) studies.

### 4.3 Cell Culture

The experiments were conducted using primary osteoblasts isolated from a patient, with a description, including the isolation protocol.^69^ The decision to select the cell line was based on the extensive use of TiO_2_-based materials for bone-related applications.^70^ Evaluating the response of osteoblasts to TFM can provide insights into their potential usage in bone regeneration, implants, or coatings for orthopedic materials. The osteoblasts were cultivated in Dulbecco’s modified Eagle medium/Ham’s nutrient mixture F12 (DMEM/F-12, GE Healthcare) supplemented with 10% fetal bovine serum (FBS, Sigma-Aldrich) and 1% penicillin–streptomycin (Pen-Strep, Sigma-Aldrich). The mixture of DMEM, FBS, and Pen-Strep is henceforth called medium for the sake of brevity. Standard growth conditions, including an incubation temperature of 37 °C and a humidified atmosphere of 5% CO_2_, were maintained during cell cultivation. Cells at passages 5–8 were used in the experiments, and the medium was refreshed every 2 days throughout the studies. Cytotoxicity evaluation was conducted at two time points (24 and 72 h) to provide insights into both early and late cell responses to TFM, respectively.

### 4.4 TFM Suspension

The synthesized material was subjected to UV light for 1 h before the experiments and tested at four various concentrations: 25, 50, 100, and 200 μg mL^–1^. Fresh batches of TFM-containing medium were prepared on the day of the experiment by making serial dilutions from a 1 mg mL^–1^ stock solution. Each concentration was tested in quintuplicate. Any remaining TFM suspensions were neither stored nor used for further experiments.

### 4.5 Cell Viability

The standard MTT assay was utilized to evaluate the viability of osteoblasts exposed to TFM. The assay relies on mitochondrial enzymes in viable cells and their ability to reduce yellow-colored MTT (3-(4,5-dimethylthiazol-2-yl)-2,5-diphenyltetrazolium bromide) into a blue formazan product. The intensity of the resultant color is proportional to the number of living cells and can be quantified using a spectrophotometer. Prior to the assay, the cells were seeded in a 96-well plate at a density of 1 × 10^4^ cells mL^–1^ and incubated for 24 h (37 °C/5% CO_2_) to allow cell attachment. Subsequently, the medium was replaced with TFM suspensions and incubated anew for the selected time periods (i.e., 24 or 72 h at 37 °C and 5% CO_2_). Cells cultured in medium only (TFM-free) served as the negative control, while the medium itself was regarded as the blank. The suspensions were removed following incubation, and a fresh medium containing 5 mg mL^–1^ MTT labeling reagent (final concentration: 0.5 mg mL^–1^) was added to each well. The plate was incubated (37 °C/5% CO_2_) for 3 h, i.e., until the formation of blue-colored formazan crystals was observed under an optical microscope. Subsequently, the wells were filled with solubilization solution (dimethyl sulfoxide), and the plate was gently shaken for approximately 5 min in the dark. Finally, the spectrophotometric absorbance was measured at 570 nm in a microplate reader (INFINITE 200 Pro). The measured absorbance values were corrected by subtracting the background signal (blank) and referenced to the negative, TFM-free control. The obtained data were verified with respect to their statistical significance by using GraphPad Prism v. 9.5 software. The differences between the analyzed probes were compared by using a two-way analysis of variance (ANOVA) with Tukey’s post hoc test. A *p*-value of <0.05 was chosen as the threshold for statistical significance. Cytotoxicity of TFM was evaluated according to the ISO 10993-5:2009 standard.

### 4.6 Cell Morphology

CLSM studies were conducted to reveal the visual effect of TFM on osteoblasts. The cells were seeded in a 96-well square glass-bottom dishes (ibidi) at a density of 1 × 10^4^ cells mL^–1^ and kept intact for 24 h (at 37 °C and 5% CO_2_) to allow attachment. The medium was then replaced with TFM suspensions and incubated again (37 °C and 5% CO_2_) for the chosen duration. The cells cultured in medium only (TFM-free) were considered the negative control. Following either the 24 or 72 h incubation, the cells were washed with phosphate-buffered saline (PBS), fixed with 3.7% paraformaldehyde solution, and permeabilized with a 0.1% solution of Triton X-100 (Sigma). 4 μL mL^–1^ Rhodamine Phalloidin (Invitrogen) and 0.5 μL mL^–1^ Hoechst 33342 (Invitrogen) were then used to stain filamentous actin and cell nuclei, respectively. Finally, threefold washing of the stained cells with PBS to clear away any dye leftovers was conducted. A Zeiss LSM880 with an Airyscan Fast module was employed to observe TFM-treated cells. Four randomly selected areas of the wells were chosen for visualization, yielding four images for each tested condition. Postprocessing of the gathered data was performed by using Zen Blue software and ImageJ open-source software.

### 4.7 Polyethylene Glycol Photodegradation

For the photodegradation of PEG under UV light irradiation, 1 mg mL^–1^ polyethylene glycol (PEG, *M*_w_ = 4000, Merck), 1 mg mL^–1^ TFM, and 0.5 wt % H_2_O_2_ (Penta chemicals) were mixed. Four control samples were prepared: (i) without adding TFM and H_2_O_2_, (ii) without UV light irradiation, (iii) without TFM, and (iv) without H_2_O_2_. All solutions were prepared in disposable cuvettes and irradiated using a 365 nm UV light-emitting diode (LED) and 450 nm visible LED for 24 h. After the photodegradation experiments, except for the control PEG solution, the other three were filtered using a membrane with a pore diameter of 220 nm. The collected solutions were stored for further analysis. PEG degradation was analyzed by MALDI-MS operated in linear positive ion detection mode. A three-layer sample preparation technique was used: a layer of 50 mg mL^–1^ *trans*-2-[3-(4-*tert*-butylphenyl)-2,2-methyl-2-propenylidene]­malononitrile in acetone in a volume of 0.2 μL dried on a stainless-steel target was overlaid with 0.2 μL of saturated solution of NaI in acetone and then with the sample solution (0.4 μL).

### 4.8 Tetracycline Photodegradation

The photocatalytic reaction was conducted in an EvoluChem PhotoRedOx TC (TC: temperature control) reactor attached to a magnetic stirrer under the illumination of a 365 nm light source. 20 mg of TFM catalyst was added to 20 mL of 10 mg L^–1^ tetracycline hydrochloride solution (Merck, ≥95%, European Pharmacopoeia HPLC assay) in the vial. Prior to photocatalysis, the mixture was agitated in the dark for about 2 h to eliminate any initial adsorption effect. Then, the photodegradation studies were carried out by irradiating the reaction mixture using a 365 nm EvoluChem UV LED 365PF nondimmable 110 V-220 V (PF Series light 18W, 365 nm 30° nondimmable 100-240VAC) and 450 nm EvoluChem LED 450PF Dimmable 220 V (PF Series 18W, 450 nm 25° Dimmable 220 V) with 328 mW cm–2 irradiance for 2 h. A 3 mL aliquot suspension was taken at regular 30 min intervals. For the scavenger experiments, 0.01 mmol EDTA, 0.02 mmol HQ, and 1 mmol IP were used as scavengers. The obtained sample was examined with a UV–visible spectrophotometer. The UV LED light spectrum and visible light spectrum are shown in Figure S8.

### 4.9 Characterization

TFM was analyzed by XRD, Raman, XPS, FESEM, EDS, and UV–vis measurements. The phase analysis was characterized by a RIGAKU SmartLab 3 kW X-ray powder diffractometer equipped with Cu Kα (λ = 1.54) radiation at 40 kV within the range of 20–80°. The Raman spectrum was measured by a Witec Alpha-300R to identify the different characteristic modes of TFM using a 532 nm laser. The chemical composition of TFM was analyzed by X-ray photoelectron spectroscopy (Kratos Analytical Axis Supra) with monochromatic Al Kα used as the excitation source. All the peaks were calibrated to the C 1s peak at 284.8 eV. The spectral fitting was done by CasaXPS software. The sample morphology, size, and elemental analysis was done using an FEI VERIOS 460L high-resolution scanning electron microscope and Talos F200i transmission electron microscopy with a FEG electron source. The FTIR spectra were recorded using a Vertex V70 spectrometer in the 4000–600 cm^–1^ wavenumber range. The UV–visible absorbance spectra were analyzed by the UV/vis/NIR spectrophotometer JascoV-770. The measurements were conducted within the wavelength range of 200–800 nm with the photomultiplier tube (PMT) detector. The absorption spectrum of TFM was obtained using the integrating sphere method, and the photocatalysis part was analyzed using rectangular cuvettes.

## Supplementary Material



## Data Availability

The authors confirm that the data supporting the findings of this study are available within the article and/or its electronic Supporting Information. The dataset for this article has been uploaded to the Zenodo repository under the DOI: 10.5281/zenodo.18339278.

## Notes

XRD of TFM-control (without PVP); SEM images of TFM-control (without PVP); TEM-EDX spectrum of TFM; HRTEM images of TFM with lattice fringes corresponding to the anatase phase and Fe_3_O_4_; XPS spectra of TFM-Control (without PVP) showing the oxidation states and splitting of metal and nonmetal ions; kinetic data of TFM under UV irradiation showing the pseudo-first-order kinetics; comparative table summarizing literature reports of PEG photodegradation; table summarizing possible fragmentation products of PEG and their actual and calculated molecular weights below 1000 Da; comparative table summarizing key literature reports of TC degradation; UV–vis spectra of TC after dark adsorption and TC after photodegradation using TFM in the presence of scavengers such as EDTA, hydroquinone, and isopropanol under UV light irradiation; spectrum of the UV and visible light sources used; and additional TEM images and SAED patterns of TFM (PDF)

## References

[1] Vattikuti, S. V. P. Heterostructured Nanomaterials: Latest Trends in Formation of Inorganic Heterostructures. In Synthesis of Inorganic Nanomaterials: Advances and Key Technologies; Elsevier, 2018; pp 89–120.

[2] Zhang, T. S. ; Yang, B. X. ; Lin, M. X. ; Zhuang, Z. Y. ; Yu, Y. Toward Rational Design of Ordered Heterostructures for Energy and Environmental Sustainability: A Review. In Advanced Energy and Sustainability Research; John Wiley and Sons Inc, 2023.

[3] Zhou Y., Zhang C., Huang D., Wang W., Zhai Y., Liang Q., Yang Y., Tian S., Luo H., Qin D. (2022). Structure Defined 2D
Mo_2_C/2Dg-C_3_N_4_ Van Der Waals Heterojunction:
Oriented Charge Flow in-Plane and
Separation within the Interface to Collectively Promote Photocatalytic
Degradation of Pharmaceutical and Personal Care Products. Appl. Catal., B.

[4] Li B., Tong F., Lv M., Wang Z., Liu Y., Wang P., Cheng H., Dai Y., Zheng Z., Huang B. (2022). In Situ Monitoring Charge Transfer on Topotactic Epitaxial Heterointerface
for Tetracycline Degradation at the Single-Particle Level. ACS Catal..

[5] Valadi Palliyalil, A. ; Ullattil, S. G. TiO_2_ Mesocrystals: Recent Progress in Synthesis, Structure, and Photocatalytic Applications. In Advanced Composites and Hybrid Materials; Springer Science and Business Media B.V., 2025.

[6] Mercyrani B., Hernandez-Maya R., Solís-López M., Th-Th C., Velumani S. (2018). Photocatalytic
Degradation of Orange G Using TiO_2_/Fe_3_O_4_ Nanocomposites. J. Mater. Sci.: Mater.
Electron..

[7] Cui Y., Zheng J., Wang Z., Li B., Yan Y., Meng M. (2021). Magnetic Induced
Fabrication of Core-Shell Structure Fe_3_O_4_@TiO_2_ Photocatalytic Membrane: Enhancing
Photocatalytic Degradation of Tetracycline and Antifouling Performance. J. Environ. Chem. Eng..

[8] Li Z., Li X., Liu C., Xue X., An J., Shi L., Xing Y., Yin D., Yoo D. J., Lyu S. (2025). Flower-like
TiO_2_ Nanostructures Coated with Fe3O4 Nanoparticles as
Magnetic Ohmic Heterojunctions for the Photocatalytic Selective Oxidative
Coupling of Primary Amines and Degradation of Tetracycline. ACS Appl. Nano Mater..

[9] Anwar M., Gumaah N. F., Ragab A. H., Taher M. A., AL-Mhyawi S. R., Gul T., Naz F., Salam T., Saeed K., Khan I. (2025). Enhancing
the Photocatalytic and Biological Potential of MnO Nanoparticles through
Strontium Doping. J. Mol. Struct..

[10] Tashkandi N. Y., Albukhari S. M., Ismail A. A. (2022). Mesoporous TiO2 Enhanced by Anchoring
Mn_3_O_4_ for Highly Efficient Photocatalyst toward
Photo-Oxidation of Ciprofloxacin. Opt. Mater..

[11] Shi X., Liu Z., Li X., You W., Shao Z., Che R. (2021). Enhanced Dielectric
Polarization from Disorder-Engineered Fe_3_O_4_@black
TiO_2-x_ Heterostructure for Broadband Microwave Absorption. Chem. Eng. J..

[12] Kubiak A. (2023). Comparative
Study of TiO_2_–Fe_3_O_4_ Photocatalysts
Synthesized by Conventional and Microwave Methods for Metronidazole
Removal. Sci. Rep..

[13] González-Anota D. E., Paredes-Carrera S. P., Pérez-Gutierrez R. M., Arciniega-Caballero B., Borja-Urby R., Sánchez-Ochoa J. C., Rojas-García E. (2023). Green Synthesis
by Microwave Irradiation of TiO_2_ Using Cinnamomum Verum
and the Application in Photocatalysis. J. Chem..

[14] Wang Y., Wen C., Hodgson P., Li Y. (2014). Biocompatibility of TiO_2_ Nanotubes with Different Topographies. J.
Biomed. Mater. Res., Part A.

[15] Vázquez-Vázquez F. C., Núñez-Tapia I. A., Guerrero-Benítez V. I., Ortiz-Magdaleno M., Chanes-Cuevas O. A., Álvarez-Pérez M. A. (2024). Multifunctional
Nanocomposite Membranes Containing TiO_2_ Developed by Air-Jet-Spun
Fibers for Tissue Engineering. Biomed. Mater.
Diagn. Devices.

[16] Kunrath, M. F. ; Farina, G. ; Sturmer, L. B. S. ; Teixeira, E. R. TiO_2_ Nanotubes as an Antibacterial Nanotextured Surface for Dental Implants: Systematic Review and Meta-Analysis. In Dental Materials; Elsevier Inc., 2024; pp 907–920. 10.1016/j.dental.2024.04.009 38714394

[17] Blendinger F., Seitz D., Ottenschläger A., Fleischer M., Bucher V. (2021). Atomic Layer Deposition of Bioactive
TiO_2_ Thin Films on Polyetheretherketone for Orthopedic
Implants. ACS Appl. Mater. Interfaces.

[18] Mackie E. J. (2003). Osteoblasts:
Novel Roles in Orchestration of Skeletal Architecture. Int. J. Biochem. Cell Biol..

[19] Jensen E. D., Gopalakrishnan R., Westendorf J. J. (2010). Regulation of Gene Expression in
Osteoblasts. BioFactors.

[20] Li K., Yan T., Xue Y., Guo L., Zhang L., Han Y. (2018). Intrinsically
Ferromagnetic Fe-Doped TiO_2_ Coatings on Titanium for Accelerating
Osteoblast Response: In Vitro. J. Mater. Chem.
B.

[21] Muthusamy S., Mahendiran B., Sampath S., Jaisankar S. N., Anandasadagopan S. K., Krishnakumar G. S. (2021). Hydroxyapatite Nanophases Augmented
with Selenium and Manganese Ions for Bone Regeneration: Physiochemical,
Microstructural and Biological Characterization. Mater. Sci. Eng., C.

[22] Wu T., Shi H., Liang Y., Lu T., Lin Z., Ye J. (2020). Improving
Osteogenesis of Calcium Phosphate Bone Cement by Incorporating with
Manganese Doped β-Tricalcium Phosphate. Mater. Sci. Eng., C.

[23] Mushtaq A., Hou Y., Tian C., Deng T., Xu C., Sun Z., Kong X., Zubair Iqbal M. (2021). Facile Synthesis
of Mn Doped TiO_2_ Rhombic Nanocomposites for Enhanced T1-Magnetic
Resonance
Imaging and Photodynamic Therapy. Mater. Res.
Bull..

[24] Zhang Z., Gu B., Zhu W., Zhu L. (2013). Integrin-Mediated Osteoblastic Adhesion
on a Porous Manganese-Incorporated TiO_2_ Coating Prepared
by Plasma Electrolytic Oxidation. Exp. Ther.
Med..

[25] Zhuang S., Wang J. (2023). Interaction between Antibiotics and Microplastics: Recent Advances
and Perspective. Sci. Total Environ..

[26] Urso M., Ussia M., Pumera M. (2021). Breaking Polymer Chains with Self-Propelled
Light-Controlled Navigable Hematite Microrobots. Adv. Funct. Mater..

[27] Lamoree M. H., van Boxel J., Nardella F., Houthuijs K. J., Brandsma S. H., Béen F., van Duursen M. B. M. (2025). Health
Impacts of Microplastic and Nanoplastic Exposure. Nat. Med..

[28] Urso M., Pumera M. (2022). Nano/Microplastics
Capture and Degradation by Autonomous
Nano/Microrobots: A Perspective. Adv. Funct
Mater..

[29] Teng C., Chen Y., Tang Z., Yuan W., Zhang L., Guo Y., Li F., Huang Q. (2024). Synthesis of Ag_2_CO_3_/TiO_2_/SiC with
pH Stability and Chloride Ion Boosted
for Efficient Photodegrading Tetracycline under Visible Light. Chem. Eng. J..

[30] Mo Y., Gao J. (2001). Polarization and Charge-Transfer Effects in Lewis Acid-Base
Complexes. J. Phys. Chem. A.

[31] Haas K. L., Franz K. J. (2009). Application of Metal
Coordination Chemistry to Explore
and Manipulate Cell Biology. Chem. Rev..

[32] Li Q., Fang G., Wu Z., Guo J., You Y., Jin H., Wan J. (2024). Advanced Microwave
Strategies Facilitate Structural
Engineering for Efficient Electrocatalysis. ChemSusChem.

[33] Sudrajat H., Babel S., Ta A. T., Nguyen T. K. (2020). Mn-Doped
TiO_2_ Photocatalysts: Role, Chemical Identity, and Local
Structure
of Dopant. J. Phys. Chem. Solids.

[34] Pishrafti H., Kamani H., Mansouri N., Hassani A. H., Ahmadpanahi H. (2022). Photocatalytic
Removal of the Erythromycin Antibiotic Using Fe-Doped TiO_2_ @Fe_3_O_4_ Magnetic Nanoparticles: Investigation
of Effective Parameters, Process Kinetics and Degradation End Products. Desalin. Water Treat..

[35] Zhao Q., Xia Z., Qian T., Rong X., Zhang M., Dong Y., Chen J., Ning H., Li Z., Hu H., Wu M. (2021). PVP-Assisted Synthesis of Ultrafine Transition Metal Oxides Encapsulated
in Nitrogen-Doped Carbon Nanofibers as Robust and Flexible Anodes
for Sodium-Ion Batteries. Carbon.

[36] Chávez-Pérez A. G., Escorcia-García J., González L. A., Reyes-Vallejo O. (2025). Tunning the Optoelectronic Properties
of Sm^3+^-Doped Mesoporous TiO_2_ Thin Films via
PVP Assisted Sol-Gel
Process for Solar Cells. J. Sol-Gel Sci. Technol..

[37] Guo H., Wang Y., Yao X., Zhang Y., Li Z., Pan S., Han J., Xu L., Qiao W., Li J., Wang H. (2021). A Comprehensive Insight
into Plasma-Catalytic Removal of Antibiotic
Oxytetracycline Based on Graphene-TiO_2_-Fe_3_O_4_ Nanocomposites. Chem. Eng. J..

[38] Norabadi E., Jahantiq A., Kamani H. (2023). Synthesis
of Fe-TiO_2_@Fe_3_O_4_ Magnetic Nanoparticles
as a Recyclable Sonocatalyst
for the Degradation of 2, 4-Dichlorophenol. Environ. Sci. Pollut. Res..

[39] Pizarro R. A. S., Herrera Barros A. P. (2020). Cypermethrin
Elimination Using Fe-TiO_2_ Nanoparticles Supported on Coconut
Palm Spathe in a Solar
Flat Plate Photoreactor. Adv. Compos. Lett..

[40] Kokorin A. I., Amal R., Teoh W. Y., Kulak A. I. (2017). Studies of Nanosized
Iron-Doped TiO_2_ Photocatalysts by Spectroscopic Methods. Appl. Magn. Reson..

[41] Zamani W., Rastgar S., Hedayati A. (2023). Capability
of TiO_2_ and
Fe_3_O_4_ Nanoparticles Loaded onto Algae (Scendesmus
Sp.) as a Novel Bio-Magnetic Photocatalyst to Degration of Red195
Dye in the Sonophotocatalytic Treatment Process under Ultrasonic/UVA
Irradiation. Sci. Rep..

[42] Alkhudaydi A. M., Danish E. Y., Lima E. C., Gabal M. A., Salam M. A. (2024). Photocatalytic
Degradation of Malachite Green Dye Using Ti_3_C_2_ MXene Nanosheets Decorated with Fe_3_O_4_ NPs
under Visible Light Irradiation. Res. Chem.
Intermed..

[43] Wahyuni E. T., Lestari N. D., Cinjana I. R., Annur S., Natsir T. A., Mudasir M. (2023). Doping TiO_2_ with Fe from
Iron Rusty Waste
for Enhancing Its Activity under Visible Light in the Congo Red Dye
Photodegradation. J. Eng. Appl. Sci..

[44] Abbas M., Parvatheeswara Rao B., Reddy V., Kim C. (2014). Fe_3_O_4_/TiO_2_ Core/Shell Nanocubes: Single-Batch Surfactantless
Synthesis, Characterization and Efficient Catalysts for Methylene
Blue Degradation. Ceram. Int..

[45] Wang W., Xiao K., Zhu L., Yin Y., Wang Z. (2017). Graphene Oxide
Supported Titanium Dioxide & Ferroferric Oxide Hybrid, a Magnetically
Separable Photocatalyst with Enhanced Photocatalytic Activity for
Tetracycline Hydrochloride Degradation. RSC
Adv..

[46] Jaiswal R., Ranganath K. V. S. (2021). Carbon Nanoparticles on Magnetite: A New Heterogeneous
Catalyst for the Oxidation of 5-Hydroxymethylfurfural (5-HMF) to 2,5-Diformoylfuran
(DFF). J. Inorg. Organomet. Polym. Mater..

[47] He W., Zhang X., Zheng K., Wu C., Pan Y., Li H., Xu L., Xu R., Chen W., Liu Y., Wang C., Sun Z., Wei S. (2023). Structural Evolution
of Anatase-Supported Platinum Nanoclusters into a Platinum-Titanium
Intermetallic Containing Platinum Single Atoms for Enhanced Catalytic
CO Oxidation. Angew. Chem., Int. Ed..

[48] Du D., Shi W., Wang L., Zhang J. (2017). Yolk-Shell Structured Fe_3_O_4_@void@TiO_2_ as a Photo-Fenton-like Catalyst
for the Extremely Efficient Elimination of Tetracycline. Appl. Catal., B.

[49] Shyniya C. R., Bhabu K. A., Rajasekaran T. R. (2017). Enhanced
Electrochemical Behavior
of Novel Acceptor Doped Titanium Dioxide Catalysts for Photocatalytic
Applications. J. Mater. Sci.: Mater. Electron..

[50] Yılmaz S., Kuyumcu Ö. K., Bayazit ŞS., Ayaz R. M. Z., Akyüz D., Koca A. (2022). Enhanced Photoelectrochemical Activity of Magnetically Modified TiO_2_ Prepared by a Simple Ex-Situ Route. J. Solid State Electrochem..

[51] Chang J., Zhang Q., Liu Y., Shi Y., Qin Z. (2018). Preparation
of Fe_3_O_4_/TiO_2_ Magnetic Photocatalyst
for Photocatalytic Degradation of Phenol. J.
Mater. Sci.: Mater. Electron..

[52] Tung W. S., Daoud W. A. (2009). New Approach toward
Nanosized Ferrous Ferric Oxide
and Fe_3_O_4_-Doped Titanium Dioxide Photocatalysts. ACS Appl. Mater. Interfaces.

[53] Kundu A., Mondal A. (2019). Structural, Optical,
Physio-Chemical Properties and
Photodegradation Study of Methylene Blue Using Pure and Iron-Doped
Anatase Titania Nanoparticles under Solar-Light Irradiation. J. Mater. Sci.: Mater. Electron..

[54] Bharti B., Kumar S., Lee H. N., Kumar R. (2016). Formation of Oxygen
Vacancies and Ti^3+^ State in TiO_2_ Thin Film and
Enhanced Optical Properties by Air Plasma Treatment. Sci. Rep..

[55] Xie W., Li R., Xu Q. (2018). Enhanced Photocatalytic
Activity of Se-Doped TiO_2_ under Visible Light Irradiation. Sci.
Rep..

[56] Shi Y., Wang C., Zhang L., Sun S. (2024). Fe-Based Deep Eutectic
Solvents for Amorphous Carbon-Encapsulated Fe_3_O_4_ Nanoparticles as Electrocatalysts for H2O2 Synthesis. ACS Appl. Nano Mater..

[57] Li H., Ma C., Wang L., Li T., Li P., Hu Y., Dai X., Wu D., Chang M., Chen Y., Xu T. (2025). 2D Black Ferroelectric
Perovskite Nanocatalysts Enable Defect Modulation-Augmented Piezocatalytic
Glioma Therapy. Adv. Funct. Mater..

[58] Li Q., Kong H., Jia R., Shao J., He Y. (2019). Enhanced Catalytic
Degradation of Amoxicillin with TiO_2_-Fe_3_O_4_ Composites: Via a Submerged Magnetic Separation Membrane
Photocatalytic Reactor (SMSMPR). RSC Adv..

[59] Jimenez-Relinque E., Llorente I., Castellote M. (2017). TiO_2_ Cement-Based Materials:
Understanding Optical Properties and Electronic Band Structure of
Complex Matrices. Catal. Today.

[60] Naldoni A., Allieta M., Santangelo S., Marelli M., Fabbri F., Cappelli S., Bianchi C. L., Psaro R., Dal Santo V. (2012). Effect of
Nature and Location of Defects on Bandgap Narrowing in Black TiO_2_ Nanoparticles. J. Am. Chem. Soc..

[61] Ullattil S.
G., Pumera M. (2023). Light-Powered
Self-Adaptive Mesostructured Microrobots
for Simultaneous Microplastics Trapping and Fragmentation via in Situ
Surface Morphing. Small.

[62] Ussia M., Urso M., Miritello M., Bruno E., Curcuruto G., Vitalini D., Condorelli G. G., Cantarella M., Privitera V., Carroccio S. C. (2019). Hybrid
Nickel-Free Graphene/Porphyrin
Rings for Photodegradation of Emerging Pollutants in Water. RSC Adv..

[63] Williams T. L., Andrzejewski D., Lay J. O., Musser S. M. (2003). Experimental Factors
Affecting the Quality and Reproducibility of MALDI TOF Mass Spectra
Obtained from Whole Bacteria Cells. J. Am. Soc.
Mass Spectrom..

[64] Wang Z., Zhang Q., Shen H., Yang P., Zhou X. (2021). Optimized
MALDI-TOF MS Strategy for Characterizing Polymers. Front. Chem..

[65] Feng Q. J., Guo S. N., Bai Z. P., Pu Y., Zhang H. T., Wang J. X. (2025). Highly Efficient Photocatalytic Degradation
of Tetracycline
Antibiotic Enabled by TiO_2_ Nanodispersion. J. Ind. Eng. Chem..

[66] Grebel J. E., Pignatello J. J., Mitch W. A. (2010). Effect of Halide Ions and Carbonates
on Organic Contaminant Degradation by Hydroxyl Radical-Based Advanced
Oxidation Processes in Saline Waters. Environ.
Sci. Technol..

[67] Chen Y., Zhang F., Guan S., Shi W., Wang X., Huang C., Chen Q. (2022). Visible Light Degradation of Tetracycline
by Hierarchical Nanoflower Structured Fluorine-Doped Bi_2_WO_6_. Mater. Sci. Semicond. Process..

[68] Yu X., He J., Zhang Y., Hu J., Chen F., Wang Y., He G., Liu J., He Q. (2019). Effective Photodegradation of Tetracycline
by Narrow-Energy Band Gap Photocatalysts La_2-X_Sr_x_NiMnO_6_ (x = 0, 0.05, 0.10, and 0.125). J. Alloys Compd..

[69] Navratilova P., Vejvodova M., Vaculovic T., Slaninova I., Emmer J., Tomas T., Ryba L., Burda J., Pavkova Goldbergova M. (2024). Cytotoxic Effects and Comparative
Analysis of Ni Ion
Uptake by Osteoarthritic and Physiological Osteoblasts. Sci. Rep..

[70] Fei
Yin Z., Wu L., Gui Yang H., Hua Su Y. (2013). Recent Progress in
Biomedical Applications of Titanium Dioxide. Phys. Chem. Chem. Phys..

