# Supplementary material for: Microwave-Driven Cytocompatible Mn-Doped TiO2–Fe3O4 Ordered Heterostructures for Microplastic and Antibiotic Degradation
Source: ACS Appl Mater Interfaces. 2026 Feb 4;18(6):10476–90. doi: 10.1021/acsami.5c20671 (PMC12926943; doi:10.1021/acsami.5c20671)
Supplement: Supplementary file 1 [file am5c20671_si_001.pdf]

Supporting Information

Microwave Driven Cytocompatible Mn Doped TiO_2-Fe_3O_4 Ordered Heterostructures for Microplastics and Antibiotic Degradation

Anjali Valadi Palliyalil,[1,2] Aiswarya Vijayakumar Thelappurath,[1,2] Daniel Wojtas,[3] Naděžda Pizúrová,[1] Monika Pávková Goldbergová,[3] Sanjay Gopal Ullattil[1]*

[1]Institute of Physics of Materials, Czech Academy of Sciences, Žižkova 513/22, 616 00, Brno, Czech Republic

[2]Central European Institute of Technology – Brno University of Technology, Purkyňova 656/123, 612 00, Brno, Czech Republic

[3]Department of Pathophysiology, Faculty of Medicine, Masaryk University, Kamenice 753/5, 625 00 Brno, Czech Republic

Corresponding Author: *ullattil@ipm.cz*

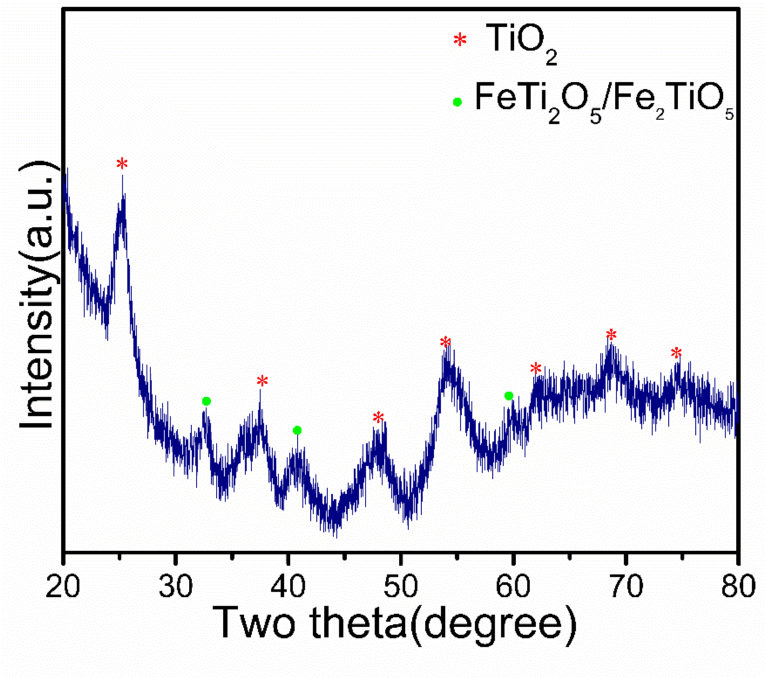

Figure S1. XRD of TFM-Control (without PVP)

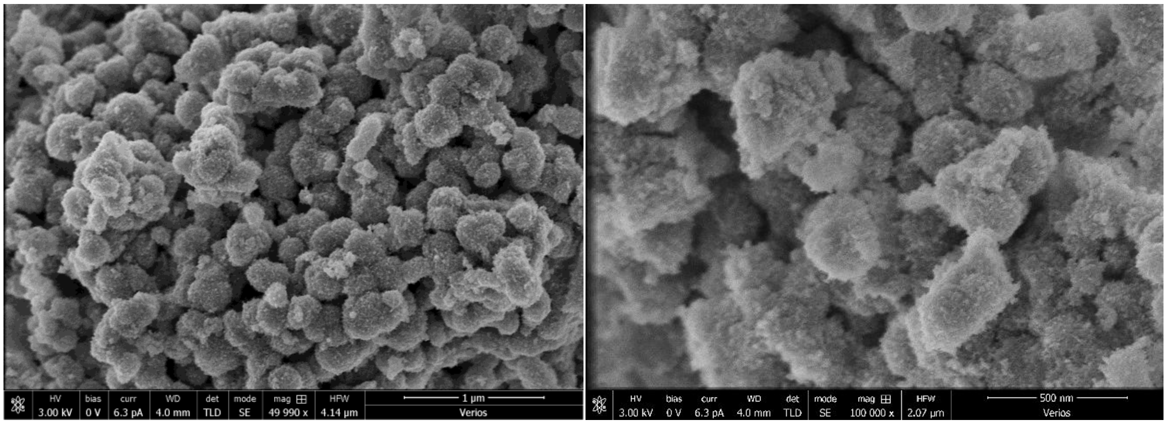

Figure S2. SEM images of TFM-Control (without PVP)

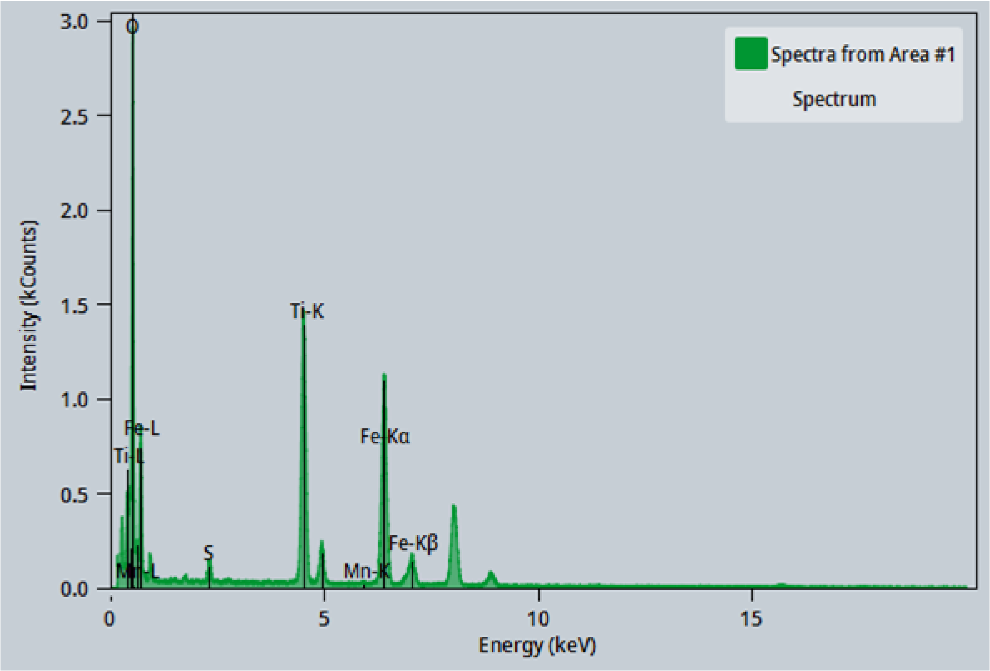

Figure S3. TEM - EDX spectrum of TFM

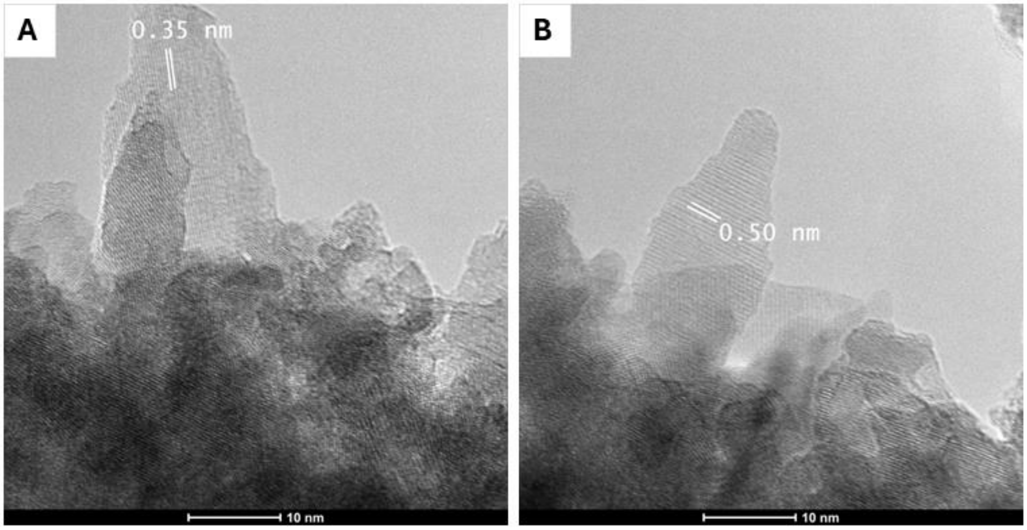

Figure S4. HRTEM images of TFM with lattice fringes corresponding to A) anatase phase B) Fe_3O_4

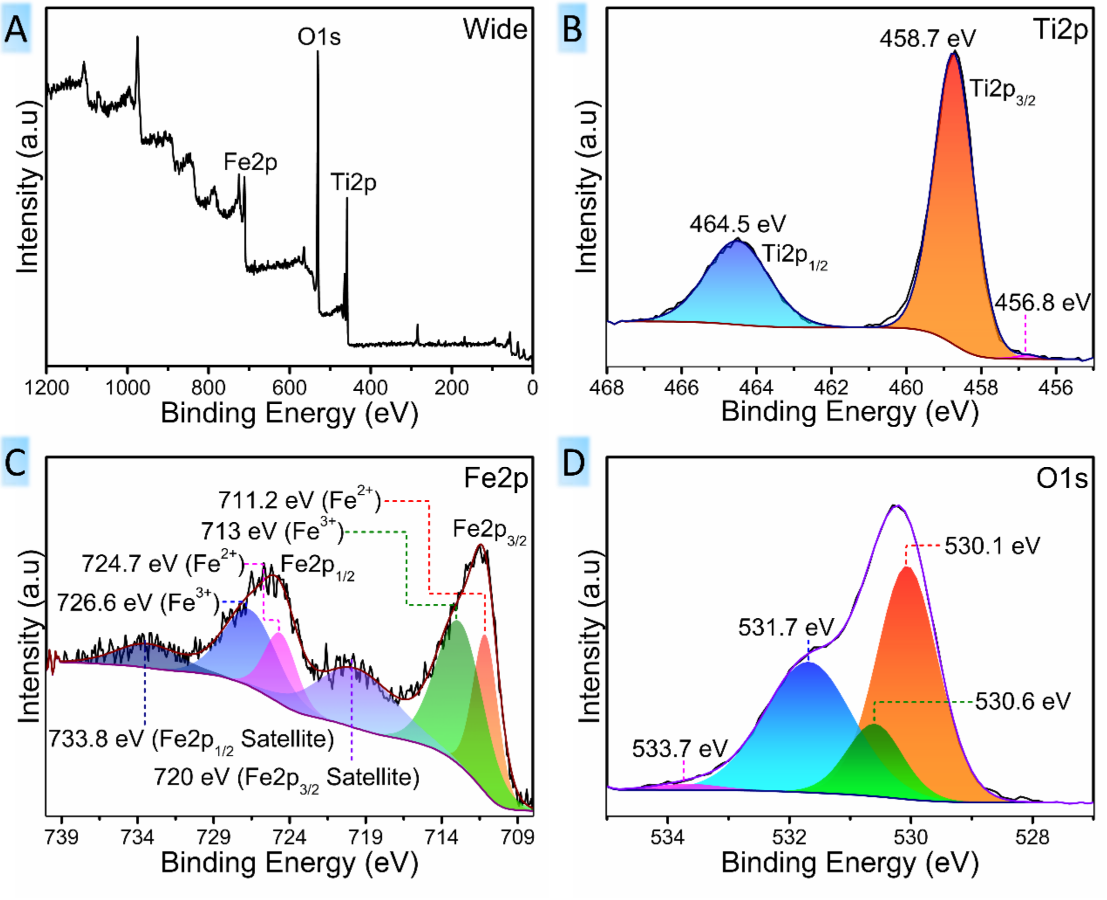

Figure S5. XPS spectra of TFM - Control (without PVP) showing the oxidation states and splitting of metal and non-metal ions, A) wide spectrum, B) Ti2p, C) Fe2p, and D) O1s

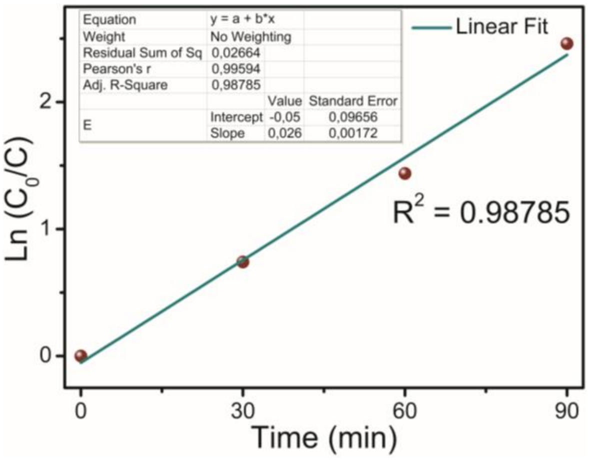

Figure S6. Kinetics data of TFM under UV irradiation showing the pseudo first order kinetics (C_0 is the initial absorbance and C is the absorbance at a specific time for TC degradation)

Table S1. A comparative table summarizing literature reports of PEG photodegradation

**Photocatalyst**	**Micro/Nanoplastic**	**Irradiation time**	**Light source**	**Reference**
Single-component TiO_2 microrobots	PEG-4000	12 h	UV	1
Hematite microrobots	PEG-4000	24 h	UV	2
Hematite/Pt Microrobots	PEG-4000	24 h	UV	3
Iron hexacyanoferrate nanobots	Nano plastics< 20 nm		Vis	4
Au@Ni@TiO_2 based micromotors	Polystyrene		UV	5

Table S2. A table summarizing possible fragmentation products of PEG and their actual and calculated molecular weight below 1000 Da

Structures	n	MNa^+ (Actual)	MNa^+ (Measured)
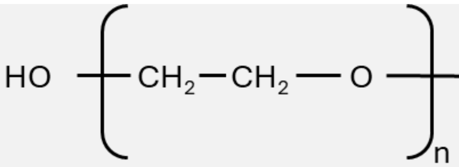	10	481.43	477
	14	657.43	653, 654
	20	921.43	918,919
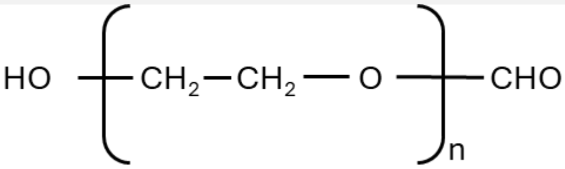	9	463.45	461
	13	639.45	639
	17	788.45	786
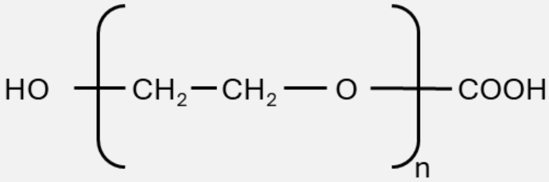	10	525.49	521
	11	569.49	565
	12	613.49	609,610
	14	701.49	697, 698
	15	745.49	742
	17	833.49	830, 831
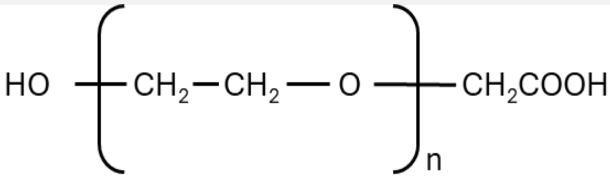	8	451.42	447
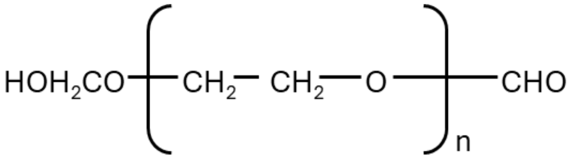			

Table S3. A comparative table summarizing key literature reports of TC degradation

**Photocatalyst**	**TC Degradation (%)**	**TC Concentration (mg/L)**	**Degradation time (min)**	**Light source**	**Ref.**
Bio templated TiO_2 UV-Fenton system	99.5	50 mg/L	40	UV	6
g-C_3N_4/TiO_2 system	96.5	50 mg/L	40	UV	7
Fe_3O_4@polydopamine/Au	98.1	20 mg/L	300	UV	8
Fe_3O_4@TiO_2 /PVDF	79.2	10 mg/L	180	Vis	9
Fe_3O_4/g-C_3N_4 /TiO_2	73.6	5mg/L	100	Vis	10
Black anatase TiO_2	66.2	10 mg/L	240	Vis	11

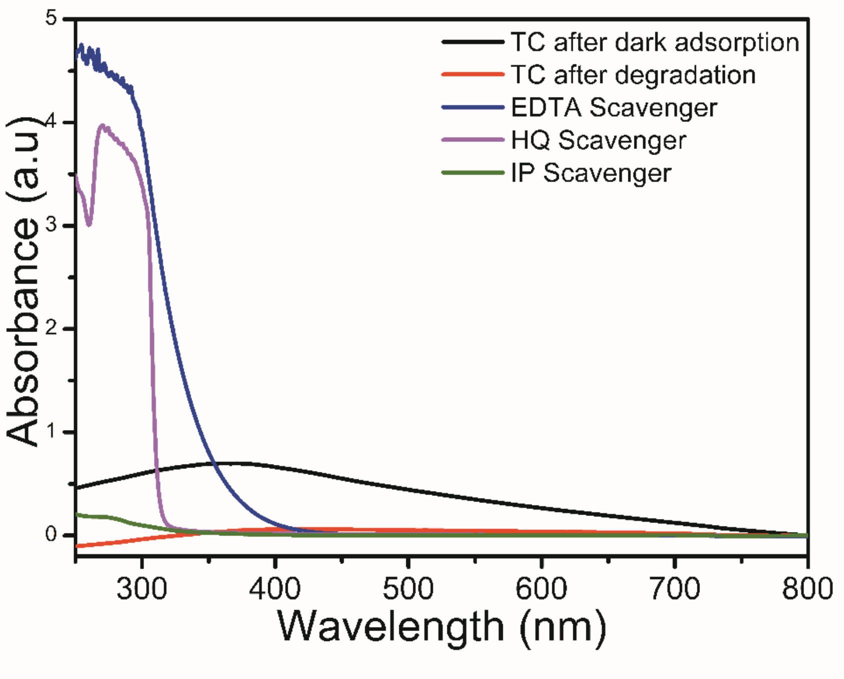

Figure S7. UV-Vis spectra of TC after dark adsorption, TC after photodegradation using TFM (90 min), TC + TFM + EDTA (90 min), TC + TFM + HQ (90 min), and TC + TFM + IP (90 min) under UV light irradiation

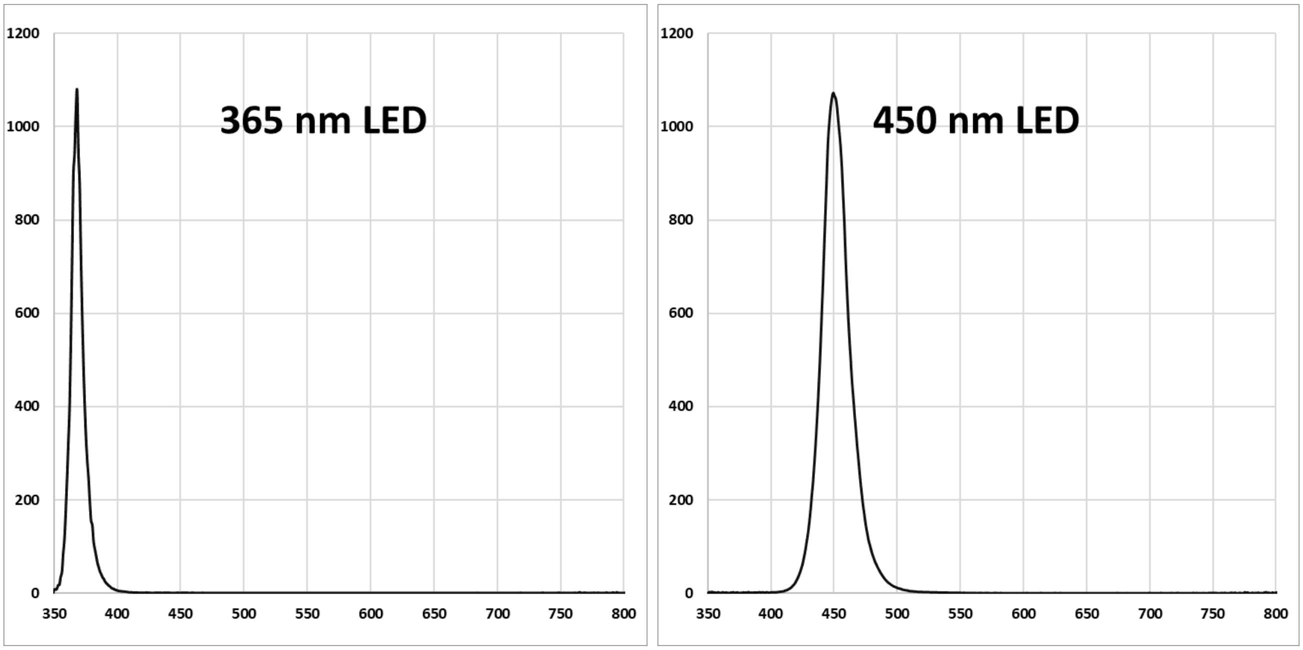

Figure S8. Spectrum of the UV and Visible light sources used

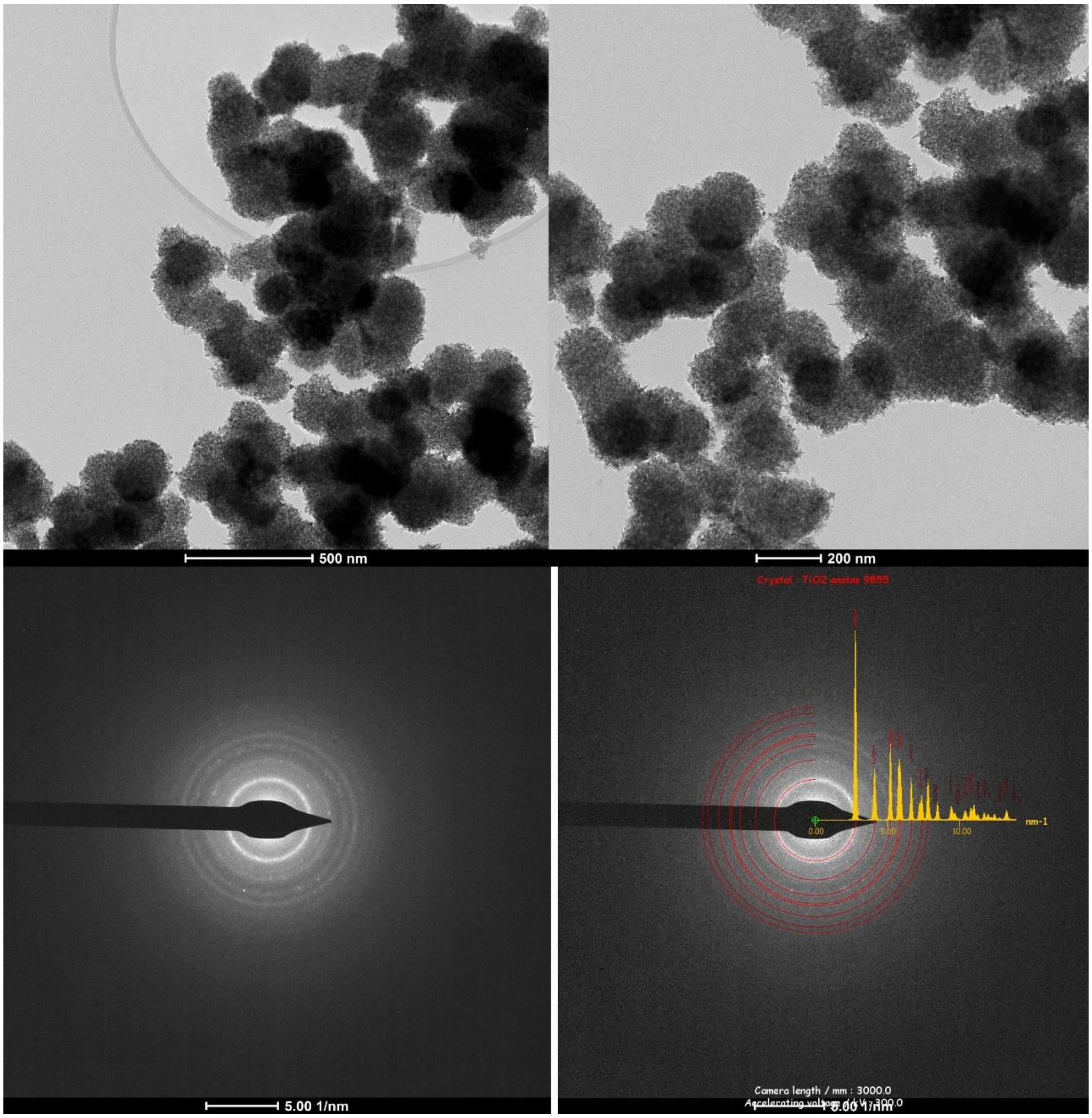

Figure S9. Additional TEM images and SAED patterns of TFM

References

1. Ullattil, S. G.; Pumera, M. Light-Powered Self-Adaptive Mesostructured Microrobots for Simultaneous Microplastics Trapping and Fragmentation via in situ Surface Morphing. *Small* **2023**, *19*(38), 2301467.
2. Urso, M.; Ussia, M.; Pumera, M. Breaking polymer chains with self-propelled light-controlled navigable hematite microrobots. *Adv. Funct. Mater.* **2021**, *31*(28), 2101510.
3. Peng, X.; Urso, M.; Ussia, M.; Pumera, M. Shape-controlled self-assembly of light-powered microrobots into ordered microchains for cells transport and water remediation. *ACS nano* **2022**, *16*(5), 7615-7625.

4. Jung, Y.; Yoon, S. J.; Byun, J.; Jung, K. W.; Choi, J. W. Visible-light-induced self-propelled nanobots against nanoplastics. *Water Res.* **2023**, *244*, 120543.
5. Wang, L.; Kaeppler, A.; Fischer, D.; Simmchen, J. Photocatalytic TiO_2 micromotors for removal of microplastics and suspended matter. *ACS Appl. Mater. Interfaces* **2019**, *11*(36), 32937-32944.
6. Yu, X.; Lin, X.; Feng, W.; Li, W. Enhanced catalytic performance of a bio-templated TiO_2 UV-Fenton system on the degradation of tetracycline. *Appl. Surf. Sci.* **2019**, *465*, 223-231.
7. Ni, S.; Fu, Z.; Li, L.; Ma, M.; Liu, Y. Step-scheme heterojunction g-C_3N_4/TiO_2 for efficient photocatalytic degradation of tetracycline hydrochloride under UV light. *Colloids Surf. A Physicochem. Eng. Asp.* **2022,** *649*, 129475.
8. Zhai, X.; Cheng, S.; Wang, H.; Zhang, C.; Li, Y.; Dong, W. Fast preparation of Fe_3O_4@ polydopamine/Au for highly efficient degradation of tetracycline. *Chemosphere* **2021**, *285*, 131523.
9. Cui, Y.; Zheng, J.; Wang, Z.; Li, B.; Yan, Y.; Meng, M. Magnetic induced fabrication of core-shell structure Fe_3O_4@ TiO_2 photocatalytic membrane: Enhancing photocatalytic degradation of tetracycline and antifouling performance. *J. Environ. Chem. Eng.* **2021** *9*(6), 106666.
10. Liu, R.; Zhang, X.; Han, X.; Sun, Y.; Jin, S.; Liu, R. J. Photocatalytic degradation of tetracycline with Fe_3O_4/g-C_3N_4/TiO_2 catalyst under visible light. *Carbon Lett.* **2024**, *34*(1), 75-83.
11. Wu, S.; Li, X.; Tian, Y.; Lin, Y.; Hu, Y. H. Excellent photocatalytic degradation of tetracycline over black anatase-TiO_2 under visible light. *Chem. Eng. J.* **2021**, *406*, 126747.
